# Posterior cingulate cortex downregulation training using fMRI neurofeedback in adolescents with early life adversity exposure: a randomized, single-blind trial

**DOI:** 10.1038/s41398-025-03445-w

**Published:** 2025-07-13

**Authors:** Xiaoqian Yu, Aki Tsuchiyagaito, Masaya Misaki, Gabe Cochran, Zsofia P. Cohen, Manpreet K. Singh, Martin P. Paulus, Robin L. Aupperle, Namik Kirlic

**Affiliations:** 1 Laureate Institute for Brain Research, Tulsa, OK USA; 2 School of Psychology, Wenzhou-Kean University, Wenzhou, ZJ China; 3 Oxley College of Health & Natural Sciences, University of Tulsa, Tulsa, OK USA; 4 Department of Psychology, Oklahoma State University, Stillwater, OK USA; 5 Department of Psychiatry and Behavioral Sciences, University of California Davis, Sacramento, CA USA; 6 School of Community Medicine, University of Tulsa, Tulsa, OK USA

**Keywords:** Human behaviour, Neuroscience

## Abstract

Early life adversity (ELA) disrupts default mode network (DMN) integrity subserving self-referential processes involved in emotional awareness and regulation. Mindfulness training (MT) reduces self-referential processing and down-regulates the DMN. We employed neurofeedback-augmented mindfulness training (NAMT), combining a core mindfulness strategy (focusing on breath) with real-time fMRI neurofeedback (rtfMRI-nf) to modulate DMN by targeting the posterior cingulate cortex (PCC). ELA-exposed (ELA; *n* = 43) and healthy control (HC; *n* = 40) adolescents completed a scan with three conditions: (a) Focus-on-breath (MT): rtfMRI-nf was presented as a variable-height bar, and adolescents attempted to lower the bar; (b) Describe: engaging self-referential processing; and (c) Rest. ELA were single-blind randomized to active PCC rtfMR-nf (NF; *n* = 22) or artificial feedback (SHAM; *n* = 21). Adolescents reported perceived stress, state mindfulness, and affect at baseline, post-training, and one-week follow-up. General linear models (GLMs) examined group differences (ELA vs. HC; NF vs. SHAM) on neural (MT vs. Describe) and self-report measures. ELA showed greater difficulty in PCC down-regulation relative to HC. For ELA, SHAM evidenced similar PCC down-regulation as active NF. All adolescents reported increased state mindfulness post-training. Relative to HC, ELA reported greater improvements in positive affect, negative affect and stress at follow-up. There was no difference in self-reported measures between active and SHAM. PCC responses in ELA confirm the region’s utility as a potential treatment target. NAMT was feasible and acceptable for ELA-exposed adolescents, but may not enhance mindfulness training more than SHAM. Optimal strategies for enhancing PCC regulation in ELA may be elucidated with future research.

## Introduction

Early life adversity (ELA), experienced by half of all children under 18 years of age in the United States [1], is characterized by abuse, neglect, household dysfunction, and peer and sibling victimization during childhood and adolescence [2]. Individuals exposed to ELA are at a dramatically increased risk for posttraumatic stress disorder (PTSD), depression, anxiety, substance use, and suicidality, as well as a host of physical health conditions including obesity, cardiovascular, gastrointestinal, and respiratory disease [3–6]. The costs of ELA exposure to society are staggering, with current yearly estimates reaching $120 billion in direct (e.g., hospitalization and mental health care) and indirect (e.g., special education and lost productivity) costs [7]. Therefore, novel treatments that are best optimized for individuals exposed to ELA are needed to address this public health crisis.

Consequences associated with ELA likely arise from closely intertwined alterations in neurobiological processes responsible for regulation of stress, including endocrine, immune, epigenetic, and brain circuitry [8, 9]. Although much of the research on the effects of trauma on the brain has focused on the disruptions in the amygdala, hippocampus, and the prefrontal cortex (PFC) [4, 10–15], recent work supports the centrality of the default mode network (DMN) [16] in the pathophysiology of ELA [17]. The DMN is a brain network active at rest and mind wandering, often involved in self-referential thinking and tasks involving judgments about personal characteristics [16, 17]. The DMN is thus thought to play a critical role in constructing our sense of self and understanding others’ perspectives [16, 17]. DMN dysfunction may contribute to difficulties in emotional awareness and regulatory control, motivation monitoring, and social cognition (e.g., theory of mind), as well as self-awareness that are often behaviorally observed in individuals with ELA [17]. Specifically, ELA may interfere with the developmental trajectory of the DMN, particularly the anterior-posterior integration of its key regions, the ventromedial PFC (vmPFC) and posterior cingulate cortex (PCC) [17, 18], contributing to emotional awareness and regulatory control difficulties. Previous studies focusing on resting-state functional connectivity in the DMN found that adults with ELA history (mean age 36 ± 10) showed decreased PCC connectivity and the MPFC/inferior temporal cortex compared to the controls without ELA history (mean age 34 ± 9) [19]. In ELA-exposed women (age 20–53 years) with PTSD, PCC activity during rest is less strongly correlated with activity in other areas of the DMN as compared to healthy controls (age 21–59 years) [18]. This is further accompanied by disrupted connectivity within the salience network (SN), such that PCC connectivity with amygdala, insula, medial PFC, and hippocampus/parahippocampal gyrus, is reduced relative to healthy controls [18]. Developmental neuroimaging studies have similarly found the associations between PTSD and reduced within-DMN connectivity in adolescents, specially, reduced PCC seeds and the middle occipital gyrus [20]. Only one study to date has shown stress impairing DMN connectivity in preadolescents. Specifically, ELA-exposed preadolescents with PTSD (age 11–13 years) had the lowest DMN theta connectivity (typical oscillatory band in this age group) in magnetoencephalography scanning during rest, indicating that stress undermines the DMN’s function as a coherent network in this population [21].

While ELA is a widespread risk factor for psychopathology affecting various aspects of life, effective treatments are scarce (see review [22]). Existing treatments, such as trauma-focused cognitive behavioral therapy, often fall short for individuals with complex trauma or severe clinical presentations (e.g., negative sense of self, affective dysregulation, and difficulties in relationships) and significant comorbidities, as these individuals tend to show less improvement in response to standard evidence-based psychotherapies [23]. Therefore, it is crucial to optimize treatments potentially by looking into the neural mechanisms underlying their effectiveness, and understanding how the treatments affect the neurocircuitry involved in ELA-related outcomes. This can help refine existing therapies and develop targeted interventions to address specific symptoms or conditions.

Mindfulness training (MT), the practice of paying attention to the present moment and viewing the current experience without judgment [24], may be well-suited for individuals exposed to ELA given that it targets key functions related to self-referential processing (e.g., DMN) [25–28]. Advanced practitioners have shown deactivation in the main nodes of the DMN [29, 30], which aligns with subjective experiences of concentration and effortless doing [31] without judgement or other related high order cognitions. Reductions in PCC activation during MT have also repeatedly been reported for participants more naïve to the practice [29, 32–34]. Thus, PCC, a key DMN node, appears to be a potential target for modulation during mindfulness practice, particularly in ELA-related psychopathology [26, 32, 35–37].

Real-time functional magnetic resonance imaging neurofeedback (rtfMRI-nf) has been successfully used to modulate brain activity related to emotional processing [38]. This technique enables individuals to gain volitional control over specific brain activity patterns by providing real-time feedback on their neural processes. During a rtfMRI-nf session, participants are trained to self-regulate brain activity in a targeted region by receiving visual or auditory feedback reflecting moment-to-moment changes in neural activation. This approach has shown promise in modifying brain function and behavior, particularly in areas such as emotion regulation, self-referential processing, and attentional control. By facilitating intentional engagement with specific neural circuits, rtfMRI-nf offers a unique tool for investigating brain-behavior relationships and holds therapeutic potential for various clinical populations.

In this study, we applied rtfMRI-nf targeting the posterior cingulate cortex (PCC) within the default mode network (DMN), which is implicated in self-referential thought and emotion regulation, to assess its impact on adolescents with early life adversity (ELA). In healthy individuals, rtfMRI-nf targeting the PCC can enhance the effects of MT on neural mechanisms involved in self-referential processing [31, 39]. Furthermore, PCC-targeted fMRI neurofeedback has been shown to improve PCC connectivity with the amygdala and anterior DMN (dmPFC, vmPFC) among adults with PTSD [40]. In a randomized controlled trial, we compared PCC-targeted rtfMRI neurofeedback-augmented mindfulness training (NAMT) responses in ELA-exposed and healthy adolescents and explored changes in PCC activity as a result of NAMT relative to healthy controls. We chose this stage of development because adolescent brains are highly plastic, allowing for more effective improvements in learning and performance, and therefore makes it a critical period to study neural correlates of psychological and behavioral strategies and their optimization [41, 42]. We predicted that adolescents exposed to ELA would exhibit greater PCC activity during NAMT compared to healthy adolescents. Moreover, adolescents exposed to ELA were randomly assigned to receive the NAMT in either an active neurofeedback condition or a SHAM condition with artificially generated feedback signal. We hypothesized that those in the active neurofeedback condition would show greater PCC down-regulation than the SHAM condition. As exploratory outcomes, we examined group differences (ELA versus HC under active neurofeedback condition; and active neurofeedback versus SHAM within ELA) in (a) percent signal change within other regions of the brain as well as PCC connectivity using whole-brain voxel-wise analysis and (b) changes in self-reported perceived stress, mindfulness, and positive/negative affect.

## Methods

### Participants

Eligible adolescents were between 13 and 17 years of age at the time of enrollment and had a parent or a legal guardian able to provide consent. HC were defined as endorsing no more than one type of maltreatment on the Maltreatment and Abuse Chronology of Exposure (MACE) scale [43] and scoring under the published cutoff scores on any subscale on the Childhood Trauma Questionnaire (CTQ) [44]. ELA endorsed four or more types of maltreatments on the MACE scale, or met the cut off score on two or more of the five subscales on the CTQ. See supplement for details on recruitment and inclusion/exclusion criteria. Seventy-five participants were included in the analysis: 34 HC (age 14.64 ± 1.19 years, 16 female) and 41 ELA (21 were randomized to the NF group [age 15.04 ± 1.32 years, 17 female], and 20 to the SHAM group [age 15.23 ± 1.33 years, 14 females]). Fig. S1 shows the CONSORT diagram.

### Experimental procedures

Study procedures were approved by Western Institutional Review Board and conducted in accordance with the Declaration of Helsinki. The study is registered at the US National Institutes of Health (ClinicalTrials.gov identifier #NCT04053582). Participants had one neurofeedback session, with questionnaires administered pre/post scan and at one week. ELA individuals were randomized via random number generator to either receive rtfMRI-nf from the PCC (NF) or artificially generated feedback signal with the similar temporal frequency characteristics as the real NF (SHAM: see Supplement). HC completed only the NF condition (data are described elsewhere [28]), leading to three groups: ELA-NF, ELA-SHAM, and HC-NF. Consensus on the Reporting and Experimental Design of clinical and cognitive-behavioral NF studies (CRED-NF checklist) [45] is in the supplement.

#### Neurofeedback augmented mindfulness training task (NAMT)

**Fig. 1:**
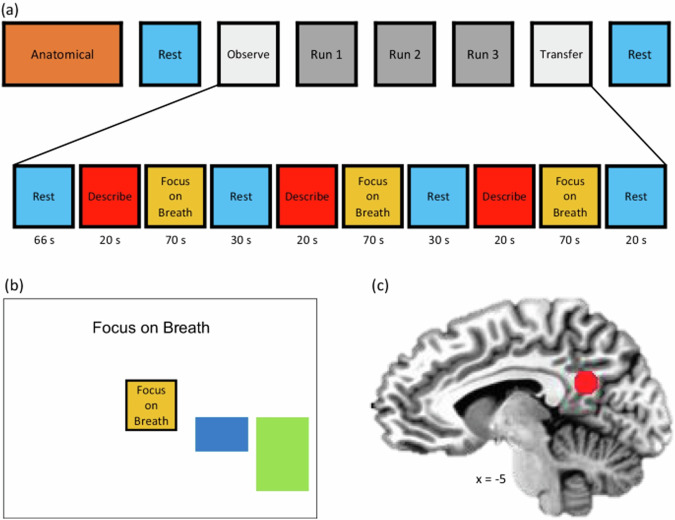
Real-time fMRI neurofeedback paradigm first published in [28]. **a** The experimental protocol consisted of eight fMRI runs, including an anatomical scan, Resting State scan 1 (Rest-1), Observe (OBS), three Neurofeedback runs (NF-1, NF-2, NF-3), a Transfer run (TRS), and Resting State scan 2 (Rest-2). During Rest runs (lasting 6 min), the participants were instructed to clear their minds and not to think about anything in particular while fixating at the display screen. OBS, NF-1, NF-2, NF-3, and TRS runs each lasted 6 min and 56 s. They started with a 66-s rest block, followed by alternating Focus-on-Breath (Mindfulness Training condition; 70 s), Describe (Active baseline condition; 20 s), and Rest (Baseline condition; 30 s) blocks. During the Focus condition, participants were instructed to pay attention to the physical sensations of their breath, not trying to change it in any way, and if their attention were to wander to something else, to gently bring it back to their breath. In the Describe condition, participants were presented with various adjectives, which they had to mentally categorize as descriptive or not descriptive of them. During the Rest condition, the participants were presented with the cue “Rest” and asked to relax while looking at the display screen. No neurofeedback was provided (no bars displayed) during the Rest and Describe conditions or during the entire OBS and TRS runs. **b** During the Focus condition, participants viewed a screen with neurofeedback bars (blue) and target bars (green). The participants were told that the blue bar may change with their experience of focusing on the breath, and that their goal to was to make the blue bar match the green bar as often as possible. The target bar remained the same height across neurofeedback runs. **c** Posterior cingulate cortex (PCC, MNI coordinates: x = −5, y = −55, z = 23) was selected as the targeted (ROI, spheres of 7-mm radius) for the real-time fMRI neurofeedback (rtfMRI-nf) training.

Prior to the scan session, adolescents were given a 30 min psychoeducational introduction into mindfulness, followed by a guided traditional MT focused on the breath (Supplement) [29, 31]. The NAMT task (Fig. 1) has been previously described [28, 46]. Briefly, the neuroimaging session included two non neurofeedback (non-NF) runs [Observe run (OBS) and Transfer run (TRS)], and three neurofeedback runs (NF-1, NF-2, NF-3). Each run lasted 6 min and 56 s, starting with a 66 s rest block (to wait for sample collection for real-time fMRI noise reduction [47]), followed by alternating Describe (active control condition; 20 s), Focus-on-Breath (MT condition with PCC neurofeedback; 70 s), and Rest (Baseline condition; 30 s) blocks. In the Describe condition, adolescents were presented with various adjectives (e.g., neat, happy), which they had to mentally categorize as descriptive or not descriptive of them (to elicit self-referential thinking) for the entire duration of word presentation. During the Focus-on-Breath condition, adolescents were instructed to attend to their physical breath-related sensations, not trying to change their breathing in any way, and to gently bring their attention back to their breath if distracted. Strategies were provided to facilitate MT prior to scanning, including “Notice the feeling of your belly rising when you breath in, and gently falling when you breath out”; “Notice if it enters and leaves through your nose or your mouth.” During the Rest condition, adolescents were presented with the cue “Rest” and asked to relax while looking at the display screen. Analyses focused on the Focus-on-Breath vs. Describe. During neurofeedback runs, participants viewed screen with neurofeedback bars and target bars (Fig. 1b). Participants were told that the blue bar may change with their experience of focusing on the breath, and that their goal was to reduce the blue bar to match the green bar as much as possible. OBS and TRS runs were identical to the NF runs, but did not involve neurofeedback (no bar displayed), that is, both essentially mindfulness conditions without feedback (See supplement for more detailed description of the NAMT task procedures). Thus, NF runs were used to evaluate the effect of NAMT, while non-NF runs were used to evaluate MT without the NF component. We expect the adolescents to be awake since after each run, adolescents had to complete the self-report for assessing aspects of feasibility and tolerability via a response box, this interaction thus kept them awake.

#### Psychological measurements

##### Task ratings

Participants completed task ratings after each fMRI run, from 1 = not at all to 10 = very much: (1) How well were you able to follow instructions on the screen? [follow instructions]; (2) How easy did you find it to focus on your breath? [focus on breath]; (3) How much did your mind wander while you were asked to focus on your breath? [mind wander]; (4) How easy did you find it to mentally decide whether or not the words described you? [describe]; (5) How easy did you find it to clear your mind while resting? [clear mind] ; and (6) How do you feel right now (from 1 = perfectly calm to 10 = very anxious)? [current feeling]. Two additional questions followed the NF runs only, from 1 = not at all to 10 = very much: (7) How well did the blue bar correspond with your experience of focusing on your breath? [blue bar corresponding with focus]; and (8) How well did the red bar correspond with the experience of your mind wandering elsewhere? [red bar corresponding with mind wander].

##### Self-reports

Participants completed the Mini-International Neuropsychiatric Interview for Children and Adolescents (MINI Kid) [48] and Patient-Reported Outcomes Measurement Information System (PROMIS) [49] at baseline. Self-reported affect, stress, and state mindfulness were measured at three timepoints: pre and post-NAMT, and one week follow-up, using the Positive and Negative Affective Schedule for Children (PANAS-C) (PANAS-C focused on state affect except for one-week follow-up, which assessed affect “during the past week”) [50], Perceived Stress Scale (PSS) [51] and the State Mindfulness Scale (SMS) including the state mindfulness of the body and state mindfulness of the mind subscales [52].

#### fMRI data acquisition and preprocessing

Neuroimaging was performed using a GE MR750 3T MRI scanner with the 8-channel receive-only head coil. For T1-weighted anatomical images, 3D magnetiza-tion-prepared rapid gradient echo (MPRAGE) pulse sequence accelerated with sensitivity encoding (SENSE) [53] was used with the following parameters: FOV/slice thickness = 240/1.2 mm, axial slices per slab = 128, image matrix size = 256 × 256, TR/TE = 5.0/1.9 ms, SENSE acceleration factor R = 2, flip angle = 8°, delay/inversion times TD/TI = 1400/725 ms, sampling band- width = 31.2 kHz, scan time = 5 min 33 s. For whole-brain fMRI recording, an accelerated single-shot gradient EPI with SENSE was used with the following parameters: FOV/slice = 240/2.9 mm, TR/TE = 2000/25 ms, SENSE ac-celeration R = 2, acquisition matrix: 96 × 96, flip angle = 90°, image matrix: 128 × 128, 46 axial slices, voxel volume: 1.9 × 1.9 × 2.9 mm^3^. Physiological pulse oximetry and respiration waveforms were recorded simultaneously with fMRI (25 ms sampling interval) using a photoplethysmograph placed on the subject’s finger and a pneumatic respiration belt. rtfMRI-nf procedures are described elsewhere [54]. Briefly, the source region of the neurofeedback signal location (aka rtfMRI-nf target) is 7-mm radius spherical, MNI coordinates: x = −5, y = −55, z = 23; Fig. 1c). The real-time fMRI signal processing included: slice-timing correction, motion correction, spatial smoothing with 6 mm-FWHM Gaussian kernel within the brain mask, scaling to a percent change relative to the average for the first 19 TRs (in the initial rest period), and regression of noise components including RETROICOR physiological noise models [55, 56] (Supplement).

Analysis of Functional NeuroImages (AFNI) (http://afni.nimh.nih.gov) [57] was used for offline data image analysis. The first 5 fMRI volumes were discarded and fMRI data preprocessing included despiking, RETROICOR [58], respiration volume per time correction [59], slice-timing and motion corrections (the cutoff for exclusion is 3 mm), nonlinear warping to the Montreal Neurological Institute (MNI) template brain with resampling to 2 mm^3^ voxels using the ANTs [60], spatial smoothing with a 6 mm FWHM Gaussian kernel, and scaling signal to percent change relative to the mean in each voxel. The signal from the PCC was calculated from a subject’s functional image using a PCC mask (MNI: −5, −55, 23; 7 mm radius sphere). The general linear model (GLM) analysis was used for independently evaluating the brain response in the OBS, NF-1, NF-2, NF-3, and TRS runs. The beta coefficient of the Focus-on-Breath block regressor was extracted to estimate brain activation during each run (OBS, NF-1, NF-2, NF-3, and TRS) and the Focus-on-Breath vs. Describe contrast was made as the primary brain outcome examining change in the PCC activity as a function of NAMT.

For PCC psychophysiological interaction (PPI) analysis, the design matrix included the PCC ROI time series orthogonalized to the PPI regressor (PCC ROI signal time series multiplied with the Focus-on-Breath block regressor [61]) in addition to the task block models, and noise regressors as described above. The volumes with >0.3-mm frame-wise displacement were censored out. Significance threshold included cluster-size correction at *p* < 0.005 voxel-wise *p* < 0.05 ( ≥55 contiguous voxels). The group analysis was performed with AFNI program 3dttest + +, and the contrast for each subject was performed with GLM (3dDeconvolve). The statistical map was thresholded voxel-wise *p* < 0.005 and cluster-size corrected *p* < 0.05 ( ≥55 contiguous voxels). The cluster size threshold was calculated by AFNI 3dClustSim with the spatial autocorrelation function.

#### Data analysis

All remaining statistical analyses were performed in R [62]. Descriptive statistics were obtained using the R package ‘psych’ [63]. Linear mixed effects models (LMEs) were conducted to examine Task Ratings across fMRI runs (OBS, NF-1, NF- 2, NF-3, and TRS) using the ‘lmer’ function in the R package ‘lme4’ [64], with Group and Run entered as fixed effects and subject ID as a random effect, while controlling for age and sex. Similarly, LMEs were conducted to examine changes in PCC BOLD signal during NF runs (averaged across three NF runs) and non-NF runs (averaged across two non-NF runs), and changes in self-report measures across different time points (before and immediately after rtfMRI-nf, and at one week follow-up). Follow-up pairwise comparisons were conducted using the “glht” function in R package [65] and corrected for multiple comparisons with the Tukey’s Honestly Significant Difference test. Effect sizes were estimated using Cohen’s d [66]. Finally, Pearson correlation coefficient was used to examine the relationship between significant covariates and outcomes of interest.

## Results

### Demographic and clinical characteristics

See Table S1 for demographic information and Table S2 for clinical characteristics.

### Participant self-reported data

#### HC-NF vs ELA-NF

**Fig. 2:**
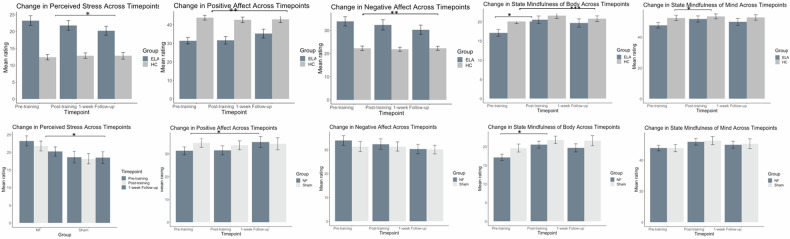
Participant reported psychological measures across timepoints: pre-training and neurofeedback, immediately post-training and neurofeedback, and at one-week follow-up. Top: HC-NF vs ELA-NF; Bottom: ELA-NF vs ELA-SHAM. The error bars represent the SE of the mean. HC healthy control, ELA early-life adversity, NF neurofeedback, OBS observe, TR transfer. *p < 0.05, **p < 0.01, ***p < 0.001.

Self-report measures. For positive affect, negative affect, and perceived stress, LME analyses revealed Group by Time interactions [positive affect*: F*_(2, 105)_ = 4.18, *p* = 0.02; negative affect*: F*_(2, 105)_ = 3.08, *p* = 0.05; perceived stress: *F*_(2, 106)_ = 4.98, *p* = 0.009]. Post-hoc analyses revealed that at one-week follow-up, ELA reported greater increases in positive affect, *p* = 0.006, as well as greater reductions in negative affect, *p* = 0.02, and perceived stress than HC, *p* = 0.002. There were main effects of Group and Time for state mindfulness of the body [Group: *F*_(1, 93)_ = 6.47, *p* = 0.01, Time: *F*_(2,106)_ = 7.49, *p* < 0.001], such that relative to ELA, HC reported higher state mindfulness of body, *p* = 0.01, and state mindfulness of body increased at post-training (*p* < 0.001) and one-week follow up (*p* = 0.01); though the Group by Time interaction was not significant [*F*_(2,106)_ = 1.91, *p* = 0.15]. There was a main effect of Time on state mindfulness of the mind [*F*_(2,106)_ = 3.26, *p* = 0.04], which increased from baseline to post-training (*p* = 0.02), but no main effect of Group [*F*_(1,72)_ = 3.73, *p* = 0.06], nor a Group by Time interaction [*F*_(2,106)_ = 1.14, *p* = 0.32] (Table 1, Fig. 2). See supplement for results concerning the impact of Age on positive affect.

**Table 1:** Unadjusted means, standard deviations, effect sizes, and main analyses of symptom measures across timepoints for healthy and ELA participants.

Measures	Estimate	SE	*t*	*p*	Cohen’s d
Perceived Stress					
Group	−10.94	1.66	−6.57	<0.001^***^	--
Timepoint					
T2	−1.43	0.85	−1.68	0.10	−0.33
T3	−3.00	0.85	−3.52	<0.001^***^	−0.68
Group * Timepoint					
T2	1.90	1.08	1.75	0.08	0.34
T3	3.41	1.08	3.15	0.002^**^	0.61
Age	−0.37	0.58	−0.63	0.53	−0.18
Sex	−0.19	1.52	−0.13	0.90	−0.04
Positive Affect					
Group	14.03	2.55	5.50	<0.001^***^	--
Timepoint					
T2	0.24	1.31	0.18	0.86	0.04
T3	3.86	1.31	2.95	0.003^**^	0.57
Group * Timepoint					
T2	−1.36	1.67	−0.81	0.42	−0.16
T3	−4.71	1.67	−2.81	0.005^**^	−0.55
Age	1.86	0.89	2.09	0.04^*^	0.58
Sex	−2.56	2.34	−1.10	0.28	−0.31
Negative Affect					
Group	−11.12	2.11	−5.27	<0.001^***^	--
Timepoint					
T2	−1.52	1.08	−1.42	0.16	−0.28
T3	−3.37	1.10	−3.08	0.002^**^	−0.60
Group * Timepoint					
T2	1.08	1.37	0.79	0.43	0.15
T3	3.37	1.38	2.44	0.02^*^	0.48
Age	−0.28	0.73	−0.38	0.71	−0.11
Sex	−1.78	1.93	−0.92	0.36	−0.26
State Mindfulness					
Group	8.74	3.93	2.22	0.03^*^	--
Timepoint					
T2	7.48	2.27	3.29	0.0013^**^	0.64
T3	4.76	2.27	2.10	0.04^*^	0.41
Group * Timepoint					
T2	−5.04	2.89	−1.74	0.08	−0.34
T3	−3.82	2.89	−1.32	0.19	−0.26
Age	1.91	1.34	1.43	0.16	0.40
Sex	−0.44	3.52	−0.13	0.90	−0.04
State Mindfulness of Body					
Group	3.20	1.26	2.54	0.01^*^	--
Timepoint					
T2	3.43	0.92	3.72	<0.001^***^	0.72
T3	2.57	0.92	2.79	0.006^**^	0.54
Group * Timepoint					
T2	−2.02	1.17	−1.72	0.09	−0.33
T3	−1.95	1.17	−1.67	0.10	−0.32
Age	0.27	0.40	0.69	0.49	0.19
Sex	−0.09	1.05	−0.08	0.93	−0.02
State Mindfulness of Mind					
Group	5.53	2.86	1.93	0.06	--
Timepoint					
T2	4.05	1.59	2.55	0.01^*^	0.50
T3	2.19	1.59	1.38	0.17	0.27
Group * Timepoint					
T2	−3.02	2.02	−1.50	0.14	−0.29
T3	−1.87	2.02	−0.93	0.36	−0.18
Age	1.63	0.98	1.66	0.10	0.47
Sex	−0.35	2.58	−0.14	0.89	−0.04

T1 Pre-training and pre-MRI, T2 Post-training and post-MRI, T3 one week follow-up.

*HC* healthy control, *ELA* early-life adversity, *NF* neurofeedback, *OBS* observe, *TR* transfer. *p < .05. **p < .01. ***p < .001.

#### ELA-NF vs ELA-SHAM

Self-report measures. LME analyses revealed main effects of Group [*F*_(1, 59)_ = 4.17, *p* = = 0.045] in perceived stress, characterized by the SHAM group reporting lower perceived stress than the NF group, *p* = 0.04. There was a main effect of Time in positive affect [*F*_(2, 76)_ = 3.35, *p* = 0.04] and state mindfulness of body [*F*_(2,78)_ = 4.59, *p* = 0.01], with positive affect being higher at one week follow-up than at baseline, *p* = 0.05, and state mindfulness of body being higher at post-training than baseline, *p* = 0.01 (Table 2, Fig. 2).

**Table 2:** Unadjusted means, standard deviations, effect sizes, and main analyses of symptom measures across timepoints for ELA participants: neurofeedback vs SHAM.

Measure	Estimate	SE	*t*	*p*	Cohen’s d
Perceived Stress					
Group	−4.40	2.15	−2.04	0.045^*^	--
Timepoint					
T2	−1.43	1.24	−1.15	0.25	−0.26
T3	−3.00	1.24	−2.42	0.02^*^	−0.55
Group * Timepoint					
T2	0.98	1.77	0.55	0.58	0.13
T3	2.85	1.77	1.61	0.11	0.36
Age	−0.19	0.73	−0.26	0.80	−0.08
Sex	−1.54	2.21	−0.70	0.49	−0.23
Positive Affect					
Group	3.22	3.05	1.06	0.29	--
Timepoint	0.24	1.67	0.14	0.89	0.03
T2	3.86	1.67	2.31	0.02^*^	0.53
T3					
Group * Timepoint					
T2	−1.39	2.39	−0.58	0.56	−0.13
T3	−3.60	2.44	−1.48	0.14	−0.34
Age	0.28	1.05	0.27	0.79	0.09
Sex	2.85	3.19	0.90	0.38	0.29
Negative Affect					
Group	−2.29	2.87	−0.80	0.43	--
Timepoint					
T2	−1.52	1.65	−0.93	0.36	−0.21
T3	−3.41	1.68	−2.03	0.045^*^	−0.46
Group * Timepoint					
T2	1.57	2.36	0.67	0.51	0.15
T3	2.07	2.40	0.86	0.39	0.20
Age	−0.95	0.98	−0.98	0.33	−0.32
Sex	−1.50	2.96	−0.51	0.62	−0.17
State Mindfulness					
Group	2.22	4.72	0.47	0.64	--
Timepoint					
T2	7.48	2.61	2.87	0.01^*^	0.65
T3	4.76	2.61	1.83	0.07	0.41
Group * Timepoint					
T2	−0.48	3.73	−0.13	0.90	−0.03
T3	−0.01	3.73	0.00	1.00	−0.0007
Age	0.33	1.62	0.20	0.84	0.07
Sex	2.24	4.90	0.46	0.65	0.15
State Mindfulness of Body					
Group	2.53	1.54	1.64	0.10	--
Timepoint					
T2	3.43	1.18	2.91	0.004^*^	0.66
T3	2.57	1.18	2.18	0.03^*^	0.49
Group * Timepoint					
T2	−1.23	1.69	−0.73	0.47	−0.17
T3	−0.62	1.69	−0.37	0.71	−0.08
Age	−0.02	0.46	−0.04	0.97	−0.01
Sex	−0.15	1.39	−0.11	0.91	−0.04
State Mindfulness of Mind					
Group	−0.31	3.47	−0.09	0.93	--
Timepoint					
T2	−0.31	3.47	−0.09	0.93	0.52
T3	4.05	1.77	2.29	0.02^*^	0.28
Group * Timepoint					
T2	0.75	2.53	0.30	0.77	0.07
T3	0.61	2.53	0.24	0.81	0.05
Age	0.35	1.21	0.29	0.78	0.09
Sex	2.40	3.68	0.65	0.52	0.21

T1 Pre-training and pre-MRI, T2 Post-training and post-MRI, T3 one week follow-up.

*HC* healthy control, *ELA* early-life adversity, *NF* neurofeedback, *OBS* observe, *TR* transfer. *p < .05.

### Posterior cingulate cortex (PCC) results

#### HC-NF vs ELA-NF

**Fig. 3:**
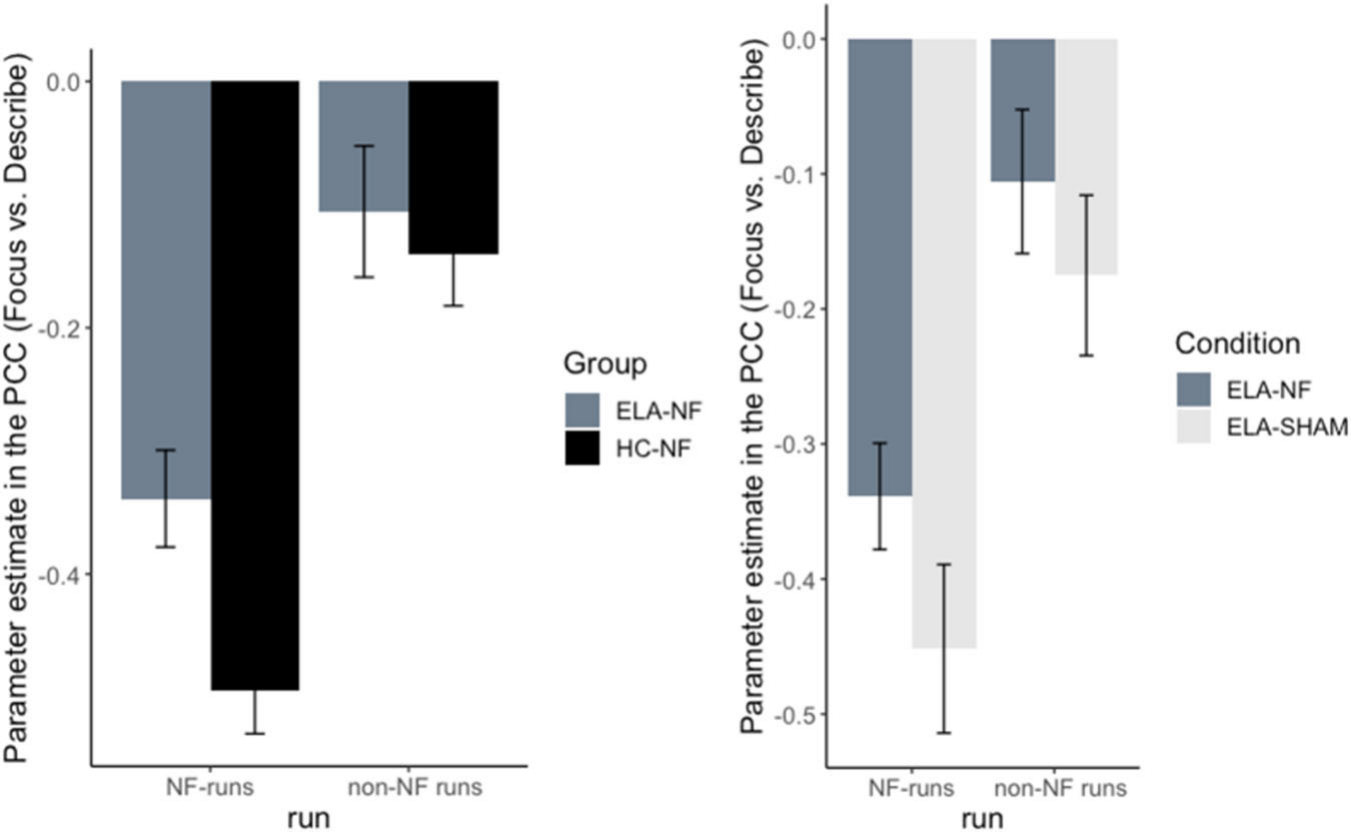
fMRI parameter estimate (Focus-on-Breath vs. Describe contrast) in the posterior cingulate cortex (PCC) in the neurofeedback (NF) runs vs non-NF runs in HC-NF and ELA-NF group (left) and ELA-NF and ELA-SHAM group (right). HC healthy control, ELA early-life adversity, NF neurofeedback, OBS observe, TRS transfer.

LME analyses revealed main effects of Group [*F*_(1, 90)_ = 4.94, *p* = 0.03] and Run [*F*_(1, 52)_ = 15.97, *p* < 0.001] for the PCC parameter estimate (Focus-on-Breath vs. Describe; Table 3, Fig. 3). Post-hoc analyses revealed that PCC activation (Focus-on-Breath vs. Describe) was lower for HC-NF than ELA-NF, *p* = 0.03 .and lower in NF than non-NF runs, *p* < 0.001. There was no Group by Run interaction [*F*_(1, 51)_ = 2.49], *p* = 0.12 (Table 4).

**Table 3:** Group, run, and interaction effects for the PCC region of interest during the Focus-on-Breath vs. Describe contrast.

HC-NF vs ELA-NF	Estimate	SE	*t*	*p*	Cohen’s d
Group	−0.15	0.07	−2.22	0.03^*^	−0.47
Run					
non-NF	0.24	0.06	4.00	<0.001^***^	1.10
Group * Run					
non-NF	0.12	0.08	1.58	0.12	0.44
Motion	0.20	0.57	0.35	0.72	0.08
Age	0.02	0.02	0.74	0.46	0.21
Sex	−0.02	0.05	−0.47	0.64	−0.13

ELA-NF vs ELA-SHAM	Estimate	SE	*t*	*p*	Cohen’s d
Group	−0.09	0.08	−1.13	0.26	−0.29
Run					
non-NF	0.24	0.06	4.14	<0.001^***^	1.32
Group * Run					
non-NF	0.04	0.08	0.50	0.62	0.16
Motion	0.63	0.82	0.77	0.44	0.18
Age	0.00	0.03	−0.09	0.93	−0.03
Sex	0.04	0.08	0.54	0.59	0.17

*PCC* posterior cingluate cortex, *HC* healthy control, *ELA* early-life adversity, *NF* neurofeedback, *OBS* observe, *TR* transfer. *p < .05. ***p < .001.

**Table 4:** PCC region of interest post hoc comparisons (Focus-on-Breath vs. Describe).

	Estimate	Std. Error	z statistic	*p* value
HC-NF vs ELA-NF				
HC vs ELA	−0.15	0.07	−2.22	0.03^*^
NF vs non-NF Run	0.23	0.06	4.00	<0.001^***^
ELA-NF vs ELA-SHAM				
NF vs non-NF Run	0.23	0.06	4.14	<0.001^***^

*HC* healthy control, *ELA* early-life adversity, *NF* neurofeedback, *OBS* observe, *TR* transfer. *p < .05. ***p < .001.

#### ELA-NF vs ELA-SHAM

There was a main effect of Run [*F*_(1,40)_ = 17.12, *p* < 0.001] in which PCC activation (Focus-on-Breath vs. Describe; Figure 6) was lower for NF than non-NF runs, *p* < 0.001; but there was no Group [*F*_(1, 59)_ = 1.28, *p* = 0.26], nor interaction effects [*F*_(1, 39)_ = 0.25, *p* = 0.62] (Table 3, Fig. 3).

### Whole brain and connectivity results

#### HC-NF vs ELA-NF

##### Whole-brain activation

Covarying for motion, age, and sex, HC showed more deactivation during neurofeedback than ELA in multiple regions including among others, the PCC, precuneus, temporal gyrus, superior medial gyrus, and fusiform gyrus (Table 5, Fig. S7).

**Table 5:** Whole-brain analysis for group differences in the mean Focus-on-Breath vs. Describe contrast during neurofeedback runs (NF-1, NF-2, NF-3).

Hemisphere/location	Peak coordinates in MNI			t	Volume (mm^3^)
ELA-NF vs HC-NF					
R Superior Temporal Gyrus	67	−25	17	3.03	430
L Calcarine Gyrus	1	−59	9	3.35	297
L Middle Temporal Gyrus	−51	−61	19	4.03	274
R Postcentral Gyrus	45	−27	41	4.62	265
L Postcentral Gyrus	−43	−37	69	3.65	227
R Fusiform Gyrus	31	−59	−17	3.37	214
L Middle Temporal Gyrus	−51	−47	7	3.74	204
L Paracentral Lobule	−1	−29	79	3.13	199
L Superior Temporal Gyrus	−61	−19	13	3.59	148
R Superior Parietal Lobule	29	−57	61	3.2	124
L Precuneus	−5	−51	75	3.02	122
L Superior Medial Gyrus	−1	41	53	3.21	120
L Superior Temporal Gyrus	−39	−23	1	3.6	98
L Precentral Gyrus	−41	3	55	3.03	92
R Cerebellum (Crus 1)	47	−65	−23	3.11	89
L Putamen	−27	−9	5	3.72	75
L Inferior Temporal Gyrus	−59	−69	−9	3.27	72
L Posterior Cingulate Cortex	−3	−41	5	3.27	69
L Caudate Nucleus	−13	−13	23	3.12	69
R Inferior Temporal Gyrus	65	−57	−9	3.85	66
L SupraMarginal Gyrus	−67	−53	25	–2.9	66
R Cerebellum (IV-V)	19	−37	−25	2.85	64
L ParaHippocampal Gyrus	−15	−33	−9	3.92	64
R Middle Temporal Gyrus	67	−19	−19	3.96	63
L Precentral Gyrus	−45	9	47	2.89	63
R Fusiform Gyrus	31	−39	−15	3.54	57
L Postcentral Gyrus	−57	−17	41	3.15	56
ELA-NF vs ELA-SHAM					
R Precuneus	7	−83	47	3.16	935
R Lingual Gyrus	21	−69	−11	3.13	389
L Fusiform Gyrus	−27	−61	−9	4.22	389
R Middle Frontal Gyrus	53	19	41	3.89	278
R Inferior Parietal Lobule	45	−51	57	3.04	217
Brain Stem	13	−23	−33	3.21	189
R Postcentral Gyrus	47	−29	43	3.68	178
R Precuneus	9	−51	37	3.04	170
L Superior Medial Gyrus	−1	57	17	−3.25	150
L Inferior Parietal Lobule	−35	−51	51	3.38	146
R Middle Temporal Gyrus	69	−23	−17	3.37	138
L Middle Frontal Gyrus	−29	37	27	–3.37	138
R Middle Temporal Gyrus	45	−55	9	2.96	121
L Middle Temporal Gyrus	−49	−71	17	2.92	86
R Lingual Gyrus	1	−69	1	2.97	65
L SupraMarginal Gyrus	−63	−23	45	2.91	57
Left Cerebellum (Crus 2)	−7	−85	−27	3.34	56

HC vs ELA (top) and NF vs SHAM (Bottom). The x, y, z coordinates indicate distance in millimeters from the anterior commissure in three dimensions: x, right to left; y, anterior to posterior; z, dorsal to ventral with positive values indicating right, anterior or dorsal and negative values left, posterior or ventral, respectively. The number of voxels in each cluster reflects contiguous voxels in which *p* < 0.000001 after applying appropriate corrections for multiple comparisons. All coordinates reported according to MNI space.

*L* left, *NF* neurofeedback run, *R* right, *HC* healthy control, *ELA* early life adversity.

##### Connectivity analysis

PCC activation during neurofeedback (Focus-on-Breath condition) was associated with many brain regions (Table 5). Compared to HC, ELA showed stronger connectivity during neurofeedback between PCC and anterior cingulate cortex (ACC), frontal gryus, precuneus, and insula, among other regions (Table 6, Fig. S8).

**Table 6:** Psychophysiological interaction (PPI) analysis (Focus-on-Breath vs. Describe contrast) for group differences during neurofeedback runs (NF-1, NF-2, NF-3).

Hemisphere/location	Peak coordinates in MNI (mm)			t	Volume (mm^3^)
ELA-NF vs HC-NF					
R Middle Temporal Gyrus	57	−21	−11	−5.42	517
R Insula Lobe	49	5	−7	5.32	286
L Precuneus	−1	−53	21	−3.01	211
R Cerebellum	3	−57	−53	–4.97	194
L Precentral Gyrus	–51	7	15	4.72	192
L Superior Frontal Gyrus	–15	59	23	4.91	141
R Cerebellum (Crus 2)	41	–75	–39	–4.13	139
R Cerebellum (VI)	17	–73	–15	–3.95	134
R Inferior Frontal Gyrus (p. Triangularis)	45	37	9	4.08	119
L Inferior Temporal Gyrus	–51	–53	–9	4.42	118
R Inferior Frontal Gyrus (p. Orbitalis)	27	37	–9	4.14	95
L Rectal Gyrus	–5	49	–21	–3.91	92
L Inferior Frontal Gyrus (p. Triangularis)	–47	35	9	3.66	87
L Middle Frontal Gyrus	–31	13	49	3.94	87
R Inferior Temporal Gyrus	53	5	–39	–4.6	83
L Anterior Cingulate Cortex	–13	31	21	5.24	81
L Cerebellum (VIII)	–21	–43	–55	–4.38	79
R Middle Temporal Gyrus	57	–43	–5	3.88	71
R Cerebellum	19	–49	–57	–4.98	70
R Precuneus	11	–55	39	–3.52	68
R Precuneus	29	–45	13	–4.55	67
L Cerebellum (X)	–9	–31	–45	–1.02	65
R Angular Gyrus	43	–69	39	–3.46	55
ELA-NF vs ELA-SHAM					
R Anterior cingulate cortex	1	51	13	4.61	679
R Angular gyrus	49	–59	29	4.51	619
R Superior medial gyrus	15	69	7	5.79	609
R Inferior frontal gyrus	43	37	11	4.62	266
R Precuneus	5	–59	35	4.53	264
R Rectal gyrus	5	37	–19	5.34	223
R Precuneus	19	–55	29	5.09	124
R Thalamus	13	–15	–3	5.48	123
L Middle Temporal Gyrus	−59	−49	−11	3.51	90
R Cerebellum (III)	9	−29	−15	4.66	88
R Middle Frontal Gyrus	25	15	43	4.75	81
L Rectal gyrus	−7	29	−15	4.88	76
L Middle Frontal Gyrus	−21	47	25	4.62	72
R Insula	45	9	−5	4.1	60
R Putamen	29	21	−1	4.2	60

Psychophysiological interaction (PPI) analysis for ELA-NF vs HC-NF (top) and ELA-NF vs ELA-SHAM (bottom). The x, y, z coordinates indicate distance in millimeters from the anterior commissure in three dimensions: x, right to left; y, anterior to posterior; z, dorsal to ventral with positive values indicating right, anterior or dorsal and negative values left, posterior or ventral, respectively. The number of voxels in each cluster reflects contiguous voxels in which *p* < 0.000001 after applying appropriate corrections for multiple comparisons. All coordinates reported according to MNI space.

*L* left, *NF* neurofeedback run, *R* right, *HC* healthy control, *ELA* early life adversity.

#### ELA-NF vs ELA-SHAM

Whole-brain activation: SHAM evidenced more deactivation during neurofeedback trials than the NF in precuneus, fusiform gyrus, frontal gyrus, temporal gyrus, among others, and less deactivation in left middle frontal gyrus and superior medial gyrus (Table 5, Fig. S7).

##### Conectivity analysis

Compared to SHAM, NF showed stronger connectivity during neurofeedback between PCC and ACC, angular gyrus, medial gyrus, frontal gryus, precuneus, insula, and putamen, among others (Table 6, Fig. S8).

## Discussion

This was the first randomized controlled trial comparing PCC-targeted rtfMRI NAMT and SHAM responses in ELA-exposed adolescents. Healthy adolescents only completed PCC-targeted rtfMRI NAMT. As we hypothesized, adolescents with ELA-exposure did not down-regulate the PCC to the same extent as the healthy adolescents, particularly during the neurofeedback runs. ELA also showed less deactivation in other regions within the DMN (e.g., precuneus) relative to HC. Surprisingly, while ELA and HC were both able to downregulate their PCC through the NAMT protocol, the impact of SHAM control was similar to PCC-targeted NF. The success in recruitment and protocol completion with 43 ELA adolescents supports the feasibility and acceptability of integrating rtfMRI and MT for this population.

When compared to healthy adolescents in both NF and non-NF runs, ELA showed attenuated PCC down-regulation that might result from a disrupted developmental trajectory of the DMN, as well as disruptions in its associated connections and functions. Previous research has indicated reduced connectivity between the DMN nodes in the PCC and the mPFC due to absent or limited anterior-posterior integration in the DMN in children before age nine, and ELA might interrupt this integration over the course of development [17]. Disruptions in the PCC and DMN has been previously linked to altered self-referential processes and emotion regulation [4, 18, 67, 68]. Disruptions in self-referential processing and emotion regulation may, in turn, pose challenges for this population to engage their PCC during the NAMT. Alternatively, more effort might be required to down-regulate the PCC in ELA generally, as ELA tend to show higher baseline PCC activity that is caused by chronic stress in early life [15]. The link between ELA and increased DMN activity might reflect difficulties disengaging from self-referential thoughts, rumination, or heightened stress responses [69]. Our findings contrast with a recent study reporting that adults with PTSD versus HC showed similar success in down-regulating the PCC during the processing of trauma-related or stressful words [70]. The difference in findings might be due to the developmental stage, or due to the strategies used during PCC regulation: the adult-focused study encouraged the use of personalized regulatory strategies while we employed a standardized mindfulness-based strategy of focusing on one’s breath to down-regulate the PCC. The discrepancy in these results may suggest that a personalized strategy (e.g., imaging biomarker guided targeting strategy) or more sessions may be more effective in supporting regulation of brain activity than a standardized strategy across everyone and across all trials [71].

Consistent with less PCC down-regulation during NF, ELA also showed less deactivation in other regions within the DMN (e.g., precuneus) relative to HC, providing further indication that ELA engaged more in self-referential processing than HC. Moreover, HC also showed more deactivation in other regions during NF than ELA, including regions involved in motor processing (precentral, postcentral, caudate, and putamen), auditory processing and social cognition process (temporal gyrus). This might suggest that HC were more focused on the task by “silencing” task-irrelevant processes. Given that ELA was also associated with greater deactivation of the supramarginal gyrus (SMG) and more active/stronger connectivity to regions that are involved in visual processing (calcarine gyrus), attention regulation and other higher level cognitive functions (superior frontal gyrus), it is possible that ELA need to exert more cognitive effort and/or engage a more distributed network to comply with NF. Notably, existing studies have found decreased connectivity within DMN in adults with ELA [14, 68, 72], while the current study found increased connectivity within DMN in adolescents with ELA relative to HC. This may be due to differences in connectivity findings for active (e.g., NF) versus rest conditions (which is used in most connectivity research) or due to developmental differences in these relationships.

The differences in PCC down-regulation between the HC and ELA groups emerged more clearly during NF runs. NF is likely a complex process that engages several higher level cognitive functions, such as monitoring, evaluating and modifying behavior in response to feedback; and these cognitive functions may be impacted by ELA [73, 74]. ELA also showed decreased connectivity among various regions in the salience network and involved in emotion regulation, including the amygdala, anterior cingulate cortex (ACC), and striatum [14, 15, 75]. Notably, the ACC plays an important role in response inhibition, error monitoring and the regulation of emotions and impulses [76]. Thus, there is the possibility that decreased connectivity between the targeted brain region (PCC) and the ACC could impair one’s ability to continually monitor success in neurofeedback and/or mindfulness training and inhibiting automatic responses in order to employ more successful regulation strategies.

Both SHAM and PCC-NF groups down-regulated the PCC more in the NF runs than non-NF runs. Although the NF group reported better correspondence between the blue bar and their experience of focusing on breath than the SHAM group (See Table S5), PCC down-regulation was comparable between the two groups. These results revealed that NF increased the effect of MT in down-regulating the PCC, regardless of whether the NF was from a specific location or a simulated random signal. This might be because similar cognitive practices/efforts were engaged in both SHAM and NF, and the SHAM group potentially continuing to modulate their PCC despite noticing the feedback did not match their experience of focusing on breath. This may indicate that prompting individuals to modulate one’s brain activity leads them to exert more effort than they would during a typical MT [77]. Moreover, some argue that the NF setup engages similar skills as mindfulness, such as the ability to focus on internal states and momentary interoceptive sensory process [78]. In particular, one study found auditory EEG-NF increased state mindfulness in adults as reflected by the increase in correct breath counts and reduced breath count resets during a brief Breath Counting Task, indicating that the NF process in and of itself might reduce the intensity or duration of mind wandering [79]. Current results approximate a prior study reporting similar improvement in depressive symptoms for both an experimental group (upregulation of emotion-related brain regions) and an active control group (upregulation of a control region activated by visual scenes) [80]. Taken together, we conclude that prompting individuals to modulate their brain activity may increase their exertion beyond typical MT practices. Moreover, the neurofeedback setup itself could foster mindfulness skills, such as focusing on internal states, reducing mind wandering and enhancing moment-to-moment sensory processing. Thus, these results align with previous findings, indicating comparable improvements in mental health outcomes irrespective of the specific or control region targeted during neurofeedback.

In whole brain analysis, SHAM showed more deactivation than NF in regions within the DMN (e.g., precuneus). We hypothesize that participants in SHAM increased their efforts by trying harder when they became aware of the discrepancy between their practice and the feedback. Relative to SHAM, NF demonstrated increased connectivity between PCC and regions responsible for attention allocation, emotion expression/regulation (ACC), and other higher cognitive functions (medial gyrus, frontal gyrus). While the functional significance of these connectivity differences are unknown, it is possible that they reflect greater engagement of cognitive control or executive functioning for regulating PCC activity during active PCC-NF. However, we note that the patterns of increased connectivity observed for NF versus SHAM were overlapping with the patterns identified for increased connectivity for ELA versus HC. It is therefore unclear whether these observed connectivity changes with NF are in the desired direction. Future research assessing the feasibility and impact of connectivity-based rtfMRI-nf could be useful for further understanding the functional role of these connectivity findings [81].

Previous studies have reported reduced DMN connectivity in both mothers and children exposed to chronic stress [21], as well as in individuals with PTSD following childhood trauma [17, 82]. In this context, the increased connectivity between precuneus and PCC in the ELA-NF group compared to the ELA-SHAM indicates that PCC-targeted NAMT enhances connectivity within the DMN. Similarly, the strengthened connectivity between the ACC and PCC in the ELA-NF group further supports the role of PCC-targeted NAMT in promoting anterior-posterior integration within the DMN, particularly between key regions such as the vmPFC and PCC [17, 18]. This finding aligns with the expected outcome, as the increased ACC-PCC connectivity observed in the ELA-NF group receiving PCC-targeted NAMT reflects the DMN connectivity restoration following targeted interventions. Moreover, our group recently examined the pre-post changes of the NAMT task on resting-state functional connectivity of the PCC in healthy adolescents, and found increase in functional connectivity between the PCC and a cluster encompassing the left hippocampus and amygdala following completion of the NAMT task, suggesting that NAMT could strengthen connectivity between the DMN and salience regions [83].

The current findings are consistent with the literature reporting that mindfulness practices involve a distributed network of regions beyond the DMN [84, 85], including the mPFC, ACC, posterior insula, hippocampus, and amygdala. This distributed network of regions reflects the complex interplay between attentional control, interoceptive awareness, and emotional regulation during MT. The observed dlPFC activation aligns with its role in implementing top-down control and managing attention [86], which is expected during mindfulness training neurofeedback (MT NF) tasks requiring sustained focus on the breath. Although there is general on-task PCC deactivation, the specific PCC deactivation linked to mindfulness appears to be uniquely associated with the subjective experience of mindfulness (i.e., reduced self-referential processing) rather than the reallocation of attention during external cognitive tasks. The Focus-on-Breath condition emphasizes present-moment awareness, potentially leading to a deliberate downregulation of DMN activity, including the PCC, as participants disengage from habitual self-referential thoughts. Moreover, it is important to highlight that ELA participants reported greater improvements in positive affect, negative affect, and stress at follow-up relative to healthy controls (HC). These therapeutic gains are unlikely to be achieved through general cognitive tasks, such as mental math or attention exercises, which do not target the same neural processes or foster the same therapeutic outcomes. Using real-time fMRI neurofeedback, prior studies have investigated the unique association and found a significant moment-to-moment correspondence between PCC activity and subjective experience of mindfulness meditation [31, 39]. However, it is important to note that these findings do not fully distinguish mindfulness meditation from other non-self-referential cognitive tasks in terms of PCC deactivation. This overlap highlights the need for further research to delineate the specific neural mechanisms underlying mindfulness meditation and their differentiation from other cognitive processes involving reduced self-referential activity.

A growing body of literature highlights the significant role of expectancy effects in shaping neurofeedback outcomes, raising important considerations for interpreting the present study’s findings. Expectancy effects refer to the influence of a participant’s beliefs and prior expectations on their response to an intervention, independent of the actual treatment mechanism. In neurofeedback, expectancy effects can be driven by factors such as the perceived sophistication of the technology, verbal cues provided by experimenters, and participants’ intrinsic motivation to regulate their neural activity. Studies have demonstrated that individuals receiving sham neurofeedback often report similar subjective improvements to those receiving real neurofeedback, suggesting that expectancy alone can drive behavioral and emotional changes [87, 88]. For example, research on sensorimotor rhythm neurofeedback in ADHD patients found that manipulating participants’ expectations about treatment efficacy significantly altered their reported symptom improvement and attentional performance, even in the absence of genuine neural modulation [89]. Additionally, meta-analyses have shown that a significant portion of neurofeedback-related cognitive and emotional improvements may be attributed to expectancy rather than direct neurophysiological effects [90]. In the present study, the comparable PCC down-regulation between SHAM and active neurofeedback conditions suggests that engagement in the task and the belief in neurofeedback efficacy may have contributed to participants’ ability to modulate self-referential processing, regardless of the actual feedback source. Addressing expectancy effects will be critical for refining neurofeedback protocols and ensuring that observed effects genuinely reflect neural self-regulation rather than non-specific therapeutic mechanisms.

### Limitations and future directions

Strengths of the current study include a sufficiently large sample size and the use of a study protocol that follows best practice recommendations set by the CRED-NF checklist (see supplement) [45]. A particularly important strength was the inclusion of a SHAM control condition, which has thus far only been included in 7% of prior rtfMRI-nf studies with clinical populations [91]. However, there were also some limitations. First, we did not measure trait mindfulness, which might impact the outcomes measured. Trait mindfulness has been found to be positively related to increases in state mindfulness following Mindfulness-based stress reduction in healthy adults [92], and negatively associated with connectivity between the nodes of the DMN [93], thus it might impact PCC down-regulation. Next, only one session of training was utilized, and thus, we were limited in our ability to examine dose effect and whether reductions in PCC activity lead to lasting improvement in self-referential processing or emotion regulation. Symptoms were found to improve in war veterans with chronic PTSD following three rtfMRI training sessions targeting the amygdala [94]. Whether changes in activity within a brain region can lead to meaningful improvement in behavioral indices of self-referential processing or emotion regulation warrants further investigation with multiple sessions. Moreover, previous studies have employed personalized ROI instead of a generic ROI [95]. Additionally, recent rtfMRI-nf research have begun to target functional connections between regions rather than activity in single ROIs [81, 96]. Future research could explore the efficacy of personalized ROI and targeting neural networks between brain regions for potentially more effective interventions. Lastly, future studies should incorporate explicit expectancy measures, such as pre- and post-intervention belief ratings, to further disentangle the specific effects of neurofeedback from broader psychological influences.

## Conclusion

This is the first study to examine the efficacy of NAMT in a single-blind, randomized, SHAM-controlled study. Results indicate that neurofeedback is feasible and acceptable for adolescents with ELA, but the PCC-target neurofeedback was not superior to a SHAM in regulating PCC activation. Comparisons with HC support the potential importance of the DMN in ELA. Thus, further identification of interventions optimized to target engagement of the PCC or the broader DMN is warranted. As an emerging technique in addressing mental health conditions, NF holds the potential to enhance treatment outcomes and shorten treatment length. Further investigation may aim to delineate the specific cognitive/affective processes and neural circuits involved in NF to maximize its benefits.

## Supplementary information

Supplementary Information: Posterior Cingulate Cortex Downregulation Training Using fMRI Neurofeedback in Adolescents with Early Life Adversity Exposure: A Randomized, Single-blind Trial

## Data Availability

The data will be made available upon reasonable request from Namik Kirlic and Martin Paulus.
