# Supplementary material for: Posterior cingulate cortex downregulation training using fMRI neurofeedback in adolescents with early life adversity exposure: a randomized, single-blind trial
Source: Transl Psychiatry. 2025 Jul 13;15:242. doi: 10.1038/s41398-025-03445-w (PMC12255819; doi:10.1038/s41398-025-03445-w)
Supplement: Supplementary file 1 — Supplementary Information: Posterior Cingulate Cortex Downregulation Training Using fMRI Neurofeedback in Adolescents with Early Life Adversity Exposure: A Randomized, Single-blind Trial [file 41398_2025_3445_MOESM1_ESM.docx]

***Supplementary Information:***

**Posterior Cingulate Cortex Downregulation Training Using fMRI Neurofeedback in Adolescents with Early Life Adversity Exposure: A Randomized, Single-blind Trial**

Xiaoqian Yu, PhD^1,2^; Aki Tsuchiyagaito, PhD^1,3^; Masaya Misaki, PhD^1^; Gabe Cochran, BS^1,4^; Zsofia P. Cohen, MS^1,4^; Manpreet K. Singh, MD, MS^5^; Martin P. Paulus^1,6^, MD; Robin L. Aupperle, PhD^1,6^; and Namik Kirlic, PhD^1^

^1^Laureate Institute for Brain Research, Tulsa, OK

^2^School of Psychology, Wenzhou-Kean University, Zhejiang, China

^3^Oxley College of Health & Natural Sciences, University of Tulsa, Tulsa, Oklahoma

^4^Department of Psychology, Oklahoma State University, Stillwater, OK

^5^Department of Psychiatry and Behavioral Sciences, University of California Davis, Sacramento, California

^6^School of Community Medicine, University of Tulsa, Tulsa, Oklahoma

**Corresponding author:**
Namik Kirlic, PhD

Affiliate Investigator

Laureate Institute for Brain Research

6655 South Yale Ave

Tulsa, OK 74136

Phone: 918-502-5747

Email: [nkirlic@laureateinstitute.org](mailto:nkirlic@laureateinstitute.org)

Keywords: Early Life Adversity Exposure; adolescents; mindfulness; fMRI neurofeedback

Table of Contents

[A Comparative Study of Active and Sham Neurofeedback in Mindfulness Training: An Intervention for Adolescents with Early Life Adversity Exposure 1](#_Toc132108146)

[Supplementary Methods 3](#_Toc132108147)

[*Recruitment* 3](#_Toc132108148)

[*Inclusion and exclusion criteria* 3](#_Toc132108149)

[*Mindfulness training (MT) prior to the neurofeedback augmented mindfulness training task (NAMT)* 3](#_Toc132108150)

[*Neurofeedback augmented mindfulness training task (NAMT)* 3](#_Toc132108151)

[*Artificially calculated feedback* 4](#_Toc132108152)

[*Fedelity rating* 5](#_Toc132108153)

[*Neuroimaging data acquisition* 5](#_Toc132108154)

[*Analyses* 5](#_Toc132108155)

[Supplementary Results 7](#_Toc132108156)

[*Participant and task-related behavioral data* 7](#_Toc132108157)

[HC-nf vs ELA-nf 7](#_Toc132108158)

[Supplementary Tables 8](#_Toc132108159)

[Supplementary Figures 26](#_Toc132108160)

# Supplementary Methods

## *Participants*

**Recruitment.** Adolescents were from a larger longitudinal study of the development and maintenance of mood, anxiety, and stress disorders in adolescents [Neuroscience-Based Mental Health Assessment and Prediction for Adolescents (NeuroMAP-A)] funded by the National Institute for General Medical Sciences Centers of Biomedical Research Excellence (CoBRE) grant^1^. Adolescents were recruited between September of 2019 and June of 2022 from the community using flyers, radio and social media advertisements, billboards, and a school-based messaging platform (i.e., PeachJar). A phone screen determined initial eligibility. Remote and in-person visits with adolescents and primary caregivers provided demographic information, medical and psychiatry history, pubertal status, family history of psychiatric illness.

**Inclusion and exclusion criteria.** Adolescents were excluded if diagnosed with a neurological or developmental disorder, were currently being managed for migraines (e.g., daily prophylactic medication), had history of traumatic brain injury, had a current psychotic disorder, bipolar disorder, obsessive-compulsive and related disorders, substance use disorder, conduct problems (i.e., conduct or oppositional defiant disorder), were currently using medications with major effects on brain function or blood flow (e.g., acne medication), and/or reported MRI contraindications. HC adolescents were free of a history of early life stress, and additionally free of (a) any lifetime or current psychiatric illness, as assessed using the Mini-International Neuropsychiatric Interview for Children and Adolescents (MINI Kid)^2^and (b) any medications with major effects on brain function or blood flow (e.g., antipsychotics, mood stabilizers, ADHD medications, SSRIs, and acne medications). A total of 83 participants were consented for the present study, with two participants withdrawn due to repeated missed appointments following consent procedures, and one participant not having usable data due to technical difficulties. Additional data from five participants were removed from analyses due to excessive head motion during MRI, leaving 75 participants in the analysis.

***Experimental Procedures***

**Mindfulness training prior to the neurofeedback augmented mindfulness training (NAMT).** Prior to NAMT, adolescents underwent a brief MT. Participants were first given a brief psychoeducational introduction into mindfulness, including that (1) mindfulness refers to paying attention to thoughts, feelings, and physical sensations in the present moment without any judgment, and (2) mindfulness can reduce stress and increase attention. Next, participants were guided through a traditional mindfulness practice focused on the breath ^3, 4^, that is: “*Please pay attention to the physical sensations of your breath where you most strongly feel it. Follow the natural and spontaneous movement of the breath, not trying to change it any way. Just pay attention to it. If you find that your attention wanders to something else, gently but firmly bring it back to the physical sensations of the breath*.” Difficulty of performing the task and how mindful they currently feel of their body and mind was assessed. Following practice, adolescents were provided with an opportunity to ask clarification questions. Next, adolescents went into the mock scanner and completed the same mindfulness practice and assessment with MRI noises in the background. Adolescents were also given instructions and feedback around minimizing motion while in the scanner. Finally, adolescents were given instructions for the neuroimaging session. Training was manualized to ensure fidelity across participants. MT was delivered by a trained research assistant under the supervision of a licensed clinical psychologist. Training sessions were audio recorded and up to 50% sessions were randomly selected for fidelity ratings by research staff. On 3-point Likert scale (0 = no adherence, 1 = adherence identified but weak or flawed, 2 = good adherence) of how closely the research assistant followed the manualized mindfulness training, the fidelity ratings indicated that the manualized training was delivered with satisfactory adherence (M = 93.25%, SD = 4.07%) (the percentage was obtained by summed score/highest total score possible). The purposes of the brief mindfulness training include: 1) ensure that participants understand the basic principles and techniques of mindfulness (e.g., focusing on the breath, 2) reduce movement and enhancing compliance, 3) simulate the mindfulness task in a controlled, non-stressful environment before the actual MRI, helping participants acclimate to the equipment and the experimental procedure, 4) ensure all participants begin the study with the same baseline level of mindfulness training, avoiding variability in prior knowledge or experience.

**Neurofeedback augmented mindfulness training (NAMT).** The neuroimaging session included 8 runs (Figure 1a), including an anatomical scan, Resting State scan 1 (Rest-1), Observe (OBS), three Neurofeedback runs (NF-1, NF-2, NF-3), Transfer run (TRS), and Resting State scan 2 (Rest-2). During each 6-minute Rest run, participants were instructed to clear their mind and not think about anything while attending to a fixation cross. OBS, NF-1, NF-2, NF-3, and TRS runs each lasted 6 minutes and 56 seconds. The initial long Rest block was required for obtaining enough samples for real-time noise regression analysis ^5^.

During the Focus-on-Breath condition^4^, adolescents were instructed to pay attention to the physical sensations of their breath, not trying to change their breathing in any way, and if their attention were to wander to something else, to gently bring their attention back to their breath^3^. To aid in MT, numerous useful strategies were provided prior to scanning, including “Notice the feeling of your belly rising when you breath in, and gently falling when you breath out”; “Notice if it enters and leaves through your nose or your mouth.” In the Describe condition, adolescents were presented with various adjectives, which they had to mentally categorize as descriptive or not descriptive of them for the entire duration the word was displayed on the screen^6^. The Describe condition is designed to elicit self-referential thinking, and therefore is thought to be a better comparator to Focus-on-Breath than Rest^3^. During the Rest condition, adolescents were presented with the cue “Rest” and asked to relax while looking at the display screen.

During neurofeedback runs, adolescents were told that they would see a bar displayed on the screen, representing the relative brain activity in a particular brain region in real time (Figure 1b). The instructions further indicated that the bar may change with the experience of focusing on the breath (i.e., the bar may go blue if they are fully concentrating on their breath, and red if their mind wanders elsewhere). The green bar represented the target to attain, and adolescents’ goal was to try and see how much they could make the bar change to blue to match the green bar. The target levels were -0.5%, -0.75%, and -1.0% (percent signal change is relative to the previous rest block) for runs NF-1, NF-2, and NF-3, respectively. In the NF condition, the bar represented the actual neurofeedback signal from PCC, which was updated continuously by changing the heigh of the bar upward or downward based on the corresponding blood-oxygen-level dependent (BOLD) activity. The bar for SHAM was artificially calculated feedback based on Fourier domain signal synthesis method. The details of the methods are explained in the later section.

Adolescents were told that there might be a 5-6 s delay between their experience and the change in the blue bar. They were asked to try their best to make the bar go blue by focusing on the physical sensations of breath.

**Artificially calculated feedback***.* A Fourier domain signal synthesis method was used to create the artificial signal for sham neurofeedback. In this approach, the real neurofeedback signal time series is transformed into the Fourier domain, and the mean and SD of the FFT series values across active healthy participants were calculated for the real and imaginary parts, respectively. Then, the random values for each value are sampled from the normal distribution with the evaluated mean and SD to create an artificial neurofeedback signal time series in the Fourier domain. Next, the inverse FFT of this artificial Fourier series gives us a synthesized neurofeedback time series. This allows us to simulate the signal with similar amplitude and temporal characteristics (e.g., autocorrelation) as the real signal.

**Fidelity rating***.* Training sessions were audio recorded and up to 50% sessions were randomly selected for fidelity ratings by research staff using an un-published 40-item measure developed by NK in consultation with RLA for the purposes of this study. Items measure whether the research assistsant (RA) gave a thorough introduction of MT (e.g., “Did RA introduce mindfulness as a coping skill”, discuss key elelments of mindfulness (e.g., “Did RA discuss non-jedgment when mind wanders”), and comply with technical details such as “Did RA turn on MRI noises for mock scan practice mindfulness”. Each item was rated on a 3-point Likert scale (0 = no adherence, 1 = adherence identified but weak or flawed, 2 = good adherence). Results showed that MT was delivered with satisfactory adherence (M = 93.25%, SD = 4.07%; percentage obtained by summed score/highest total score possible).

**Neuroimaging data acquisition**. Neuroimaging was performed using a GE MR750 3T MRI scanner with the 8-channel receive-only head coil. For T1-weighted anatomical images, 3D magnetiza-tion-prepared rapid gradient echo (MPRAGE) pulse sequence accelerated with sensitivity encoding (SENSE) ^7^ was used with the following parameters: FOV/slice thickness = 240/1.2 mm, axial slices per slab = 128, image matrix size=256 × 256, TR/TE = 5.0/1.9 ms, SENSE acceleration factor R = 2, flip angle = 8°, delay/inversion times TD/TI = 1,400/725 ms, sampling band- width = 31.2 kHz, scan time = 5 min 33 s.

For whole-brain fMRI recording, an accelerated single-shot gradient EPI with SENSE was used with the following parameters: FOV/slice = 240/2.9 mm, TR/TE = 2000/25 ms, SENSE ac-celeration R=2, acquisition matrix: 96 × 96, flip angle = 90°, image matrix: 128 × 128, 46 axial slices, voxel volume: 1.9 × 1.9 × 2.9 mm3. Physiological pulse oximetry and respiration waveforms were recorded simultaneously with fMRI (25 ms sampling interval) using a photoplethysmograph placed on the subject's finger and a pneumatic respiration belt. rtfMRI-nf procedures are described elsewhere^8^.

**Psychological measurements**

***Task ratings****.* To assess for aspects of feasibility and tolerability, adolescents answered the following questions via a response box and visual analog scale after reach run, from 1=not at all to 10= very much: (1) How well were you able to follow instructions on the screen? [follow instructions]; (2) How easy did you find it to focus on your breath? [focus on breath]; (3) How much did your mind wander while you were asked to focus on your breath? [mind wander]; (4) How easy did you find it to mentally decide whether or not the words described you? [describe]; (5) How easy did you find it to clear your mind while resting? [clear mind] ; and (6) How do you feel right now (from 1 = perfectly calm to 10 = very anxious)? [current feeling]. Two additional questions followed the NF runs only: (7) How well did the blue bar correspond with your experience of focusing on your breath? [blue bar corresponding with focus]; and (8) How well did the red bar correspond with the experience of your mind wandering elsewhere? [red bar corresponding with mind wander].

## *Analyses*

**Real-time fMRI processing.** The region of interest (ROI) and rtfMRI-nf target location (spherical ROI, 7mm radius, [MNI coordinates: x = -5, y=-55, z = 23]; Figure 1a) was selected based on a meta-analysis investigating functional neuroimaging studies of the DMN ^9^, mindfulness meditation studies, including neurofeedback ^3, 4^, and conducted pilot testing.

We employed an advanced fMRI real‐time processing (RTP) protocol which included: slice‐timing correction, motion correction, spatial smoothing with 6 mm‐FWHM Gaussian kernel within the brain mask, scaling to a percent change relative to the average for the first 19 TRs (in the initial rest period), and regressing out noise components ^5, 10^. The noise regressors were six motion parameters, eight RETROICOR ^11^ regressors (four cardiac and four respiration), white matter mean signal, ventricle mean signal, and Legendre polynomial models of slow signal fluctuation. This comprehensive noise reduction was performed in real‐time (less than 400 ms ^5^). This fMRI RTP system operates real-time motion tracking, alignment, and motion parameter regression, thus allowing for suppression of head motion effects, and importantly, providing physiological noise correction (RETROICOR) in real time before the PCC based neurofeedback signal computation and visual presentation to the adolescent ^5, 12, 13^. This ensured that PCC neurofeedback signal reflected the largest possible extent the underlying neuronal activity and does not reflect head motion, heart rate, and/or respiratory motions, all of which overlap with DMN neural activity ^14, 15^. After real-time noise regression, the fMRI RTP system exports the mean value of the noise-reduced signals for the PCC ROI for each acquired data volume.

The PCC ROI (i.e., neurofeedback target signal) in the MNI space was warped into the individual brain space using the Advanced Normalization Tools (ANTs) software ^16^ (http://stnava.github. io/ANTs/). The neurofeedback stimulus was delivered via custom-developed software using PsychoPy ^17^. The neurofeedback value was a signal change relative to the baseline obtained by averaging the preceding 30 s long Rest condition. The two initial volumes in the Rest condition were excluded from the baseline calculation to avoid the delayed hemodynamic response effect of the preceding Describe block. The neurofeedback started from the third volume in the Focus block to wait for a hemodynamic response delay. The bar height was updated at every TR as a moving average of the current and up to the two available preceding values to reduce the bar fluctuation ^18^.

**Offline data processing.** The first 5 fMRI volumes were discarded and fMRI data preprocessing included despiking, RETROICOR^11^, respiration volume per time correction^19^, slice-timing and motion corrections, nonlinear warping to the Montreal Neurological Institute (MNI) template brain with resampling to 2 mm^3^ voxels using the ANTs^16^, spatial smoothing with a 6 mm FWHM Gaussian kernel, and scaling signal to percent change relative to the mean in each voxel. General linear model (GLM) analyses were used for independently evaluating the brain response for OBS, NF-1, NF-2, NF-3, and TRS runs. The design matrix included a modeled response to the Focus-on-Breath block (boxcar function convolved with hemodynamic response function), 12 motion parameters (3 shift and 3 rotation parameters with their temporal derivatives), three principal components of the ventricle signal, local white matter average signal (ANATICOR)^20^, and low-frequency fluctuations (fourth-order Legendre polynomial model). The beta coefficient of the Focus-on-Breath block regressor was extracted to estimate brain activation during each run (OBS, NF-1, NF-2, NF-3, and TRS) and the Focus-on-Breath vs. Describe (the run immediately prior) contrast was caluculate as the primary brain outcome analysis examining change in the PCC activity as a function of NAMT.

**Physiologial noise correction.** Our study is one of the first to include real-time physiological noise correction for the rtfMRI-nf^21^; we also used offline RETROICOR correction in our preprocessing pipeline and further physiological noise correction during the subsequent GLM analysis. To evaluate the efficacy of physiological noise correction, we calculated the signal variance ratio (R^2 value) explained by the physiological noise regressors (RETROICOR) for the real-time processed and offline processed signals in the PCC region. This measure estimates the amount of residual physiological noise in the processed signal^21^. The R^2 was small (< 0.25) for both real-time and offline-processed signals on average (Figure S2). While some subjects' runs showed a relatively high residual noise variance ratio, there was no significant correlation between the mean neurofeedback signal amplitude and the residual noise variance ratio for the real-time processed data (Spearman's *rho* = -0.091, *p* = 0.361). Although a relatively high residual physiological noise variance ratio was seen for the offline-processed data, the correlation between the PCC parameter estimates (beta value) and the physiological noise variance ratio was not significant for the offline-processed data (Spearman's *rho* = 0.031, *p* = 0.744). These indicate that the physiological noise effect was not significant on the neurofeedback signal in the real-time analysis and the PCC parameter estimation in the offline analysis.

# Supplementary Results

## *Participant and task-related behavioral data*

## HC-NF vs ELA-NF

*Task Ratings.* For the describe and current feeling ratings, LME analyses revealed a main effect of Group [describe: *F*_(1, 110)_ = 5.21, *p* = .02; feeling: *F*_(1, 115)_ = 15.10, *p* < .001], but not Run [describe: *F*_(4, 179)_ = 0.54, *p* = .70; feeling: *F*_(4, 179)_ = 1.97, *p =*.10], nor Group by Run interaction [describe: *F*_(4, 179)_ = .86, *p* = .50 ; feeling: *F*_(4, 179)_ = 1.08, *p* = .37] (Table S1). Post-hoc analyses showed that relative to ELA, HC reported greater ease of mentally deciding whether words described them during the Describe condition (*p* = .02), and feeling more calm than ELA (*p* < .001) across all runs. Additionally, age was a significant covariate for self-reported affect

[feeling: *F*_(1, 46)_ = 5.23, *p* = .03], such that relative to younger adolescents, older adolescents reported feeling more calm [*r_53_* = -0.18, *p* = .005]. For the rating of ability to follow task instruction, there was a main effect of Run [*F*_(4, 180)_ = 4.14, *p* = .003], such that adolescents were better at following instructions in NF-1 (*p* = .002) and NF-2 (*p* = .02) than OBS. There were no main effect of Group [*F*_(1, 97)_ = 3.78, *p* = .055] or Group by Run interaction [*F*_(4, 179)_ = 1.15, *p* = .33] (Table S3, Figure S3).

*Self-report measures.*LME analyses revealed main effects of Group and Time for positive affect [Group: *F*_(1, 68)_ = 30.19, *p* <.001; Time [*F*_(2, 105)_ = 5.44, *p* = .006], negative affect [Group: *F*_(1, 68)_ = 27.8, *p* <.001; Time [*F*_(2, 105)_ = 4.74, *p* = .01], and perceived stress [Group: *F*_(1, 68)_ = 43.20, *p* <.001; Time [*F*_(2, 106)_ = 6.21, *p* = .003]. All were qualified by Group by Time interaction [positive affect*: F*_(2, 105)_ = 4.18, *p* = .02; negative affect*: F*_(2, 105)_ = 3.08, *p* = .05; perceived stress: *F*_(2, 106)_ = 4.98, *p* = .009]. Post-hoc analyses revealed that at one-week follow-up, ELA reported increased positive affect, *p =* .006, as well as reduced negative affect, *p =* .02, and perceived stress than HC, *p =* .002. Additionally, age was a significant covariate in self-reported positive affect [*F*_(1, 51)_ = 4.36, *p* = .04], such that older adolescents reported higher levels of positive affect than younger adolescents [*r(_53)_* = .16, *p* = .046]. There were main effects of Group and Time for state mindfulness of the body [Group: *F*_(1, 93)_ = 6.47, *p* = .01, Time: *F*_(2,106)_ = 7.49, *p* <.001], such that relative to ELA, HC reported higher state mindfulness of body, *p* = .01, and state mindfulness of body increased at post-training (*p* <.001) and one-week follow up (*p =* .01). This was not qualified by Group by Time interaction [*F*_(2,106)_ = 1.91, *p =* .15]. There was a main effect of Time on state mindfulness of the mind [*F*_(2,106)_ = 3.26, *p* = .04], which increased from baseline to post-training (*p* = .02). There was not a main effect of Group [*F*_(1,72)_ = 3.73, *p* = .06], nor Group by Time interaction [*F*_(2,106)_ = 1.14, *p* = .32] (Table S3, Figure S3).

**ELA-NF vs ELA-SHAM**

*Task Ratings.* LME analyses revealed a main effect of Group [*F*_(1, 78)_ = 4.12, *p* = .045] and Time [*F*_(4, 95)_ = 2.91, *p* = .03] in the ease of following instruction, but not Group by Time interaction [*F*_(4, 95)_ = 1.00, *p* = .41] (Table S2). Post-hoc analyses revealed that the NF group reported more difficulty in following instructions than the SHAM group, and were easier to follow instructions in NF-1 than OBS. There was a main effect of Group for “how well the blue bar corresponds with experience of focusing on breath” [*F*_(1, 65)_ = 6.47, *p* = .01], but not Time [*F*_(2,48)_ = .2, *p* = .81], nor Group by Time interaction [*F*_(2,47)_ = .42, *p* = .66]. NF group reported that blue bar corresponded better with their experience of focusing on breath than the SHAM group, *p* = .01 (Table S4, Figure S4).

*Self-report measures.* LME analyses revealed main effects of Group [*F*_(1, 59)_ = 4.17, *p* = .045] in perceived stress, but not Time [*F*_(2,78)_ = 2.94, *p* = .06], nor Group by Time interaction [*F*_(2,78)_ = 1.33, *p* = .27]. Post-hoc analyses revealed that the SHAM group reported lower perceived stress than the NF group, *p* = .04. There was a main effect of Time in positive affect [*F*_(2, 76)_ = 3.35, *p* = .04] and state mindfulness of body [*F*_(2,78)_ = 4.59, *p* = .01]; but not Group [positive affect: *F*_(1,56)_ = 1.12, *p* = .29; state mindfulness of body: *F*_(1,84)_ = 2.69, *p* = .10], nor Group by Time interaction [positive affect: *F*_(2,76)_ = 1.10, *p* = .34; state mindfulness of body: *F*_(2,78)_ = .26, *p* = .77]. Post-hoc analyses indicated that positive affect at one week follow-up was higher than that at baseline, *p* = .05, and post-training state mindfulness of body was higher than baseline, *p* = .01 (Table S4, Figure S4).

**Additional analyses on the PCC activity**

HC-NF vs ELA-NF. LME analyses revealed main effect of Run [*F*_(4, 210)_ = 5.27, *p* <.001] in PCC activity for the Focus-on-Breath vs. Describe contrast (Fig 1, top). Post-hoc analyses revealed that PCC activity in NF1, NF2 was smaller than OBS (*p* <.01 for NF-1 and *p* <.05 for NF-2) and TRS (*p* < .05). (Figure S5 , top).

ELA-NF vs ELA-SHAM. LME analyses revealed main effects of Run [*F*_(4, 155)_ = 3.97, *p* <.01] for parameter estimate (Focus-on-Breath vs. Describe) in the PCC (Figure S5, bottom). Post-hoc analyses revealed that PCC activity in NF1, NF2 was smaller than OBS, *p* <.05.

**Analyses of the PCC activity in Describe task**

HC-NF vs ELA-NF. There is no main effects of Group [*F*_(4, 210)_ = 2.4, *p* = .12] , but the main effect of Run is trending [*F*_(4, 210)_ = 3.74, *p* = .06] (Figure S6, left). Although the results do not reach a significance, it is puzzling that PCC activity appears to increase in HC during the NF when compared to the ELA.

ELA-NF vs ELA-SHAM. There is no main effects of Group [*F*_(4, 155)_ = 1.47, *p* = .22] , but the main effect of Run is trending [*F*_(4, 155)_ = 3.75, *p* = .06] for parameter estimate in the PCC (Figure S6, right). The PCC activity in Describe appears to decrease in ELA-NF than the ELA-SHAM, suggesting the potential effect of PCC-targeted NF on PCC.

Since self-referential process involves a complex set of cognitive functions that potentially engage a distributed network^34^, and the whole brain analysis also showed that ELA showed less deactivation in other regions within the DMN (e.g., precuneus). Together these findings suggest that a generic ROI might not be optimal for rtfMRI-NF. A personalized ROI or functional connections between brain regions might be more ideal targets for rtfMRI-NF design that intended to engage self-referential processing.

# Supplementary Tables

Table S1. Demographic information for HC-NF, ELA-NF, and ELA-SHAM.

| Demographic | | HC-NF(n=34) | ELA-NF(n=21) | ELA-SHAM (n=20) | HC-NF vs ELA-NF | | ELA-NF vs ELA-SHAM | |
| --- | --- | --- | --- | --- | --- | --- | --- | --- |
|  |  | |  |  | *Fisher’s exact* | *p* | *Fisher’s exact* | *p* |
| Sex |  | |  |  |  |  |  |  |
| Male | 18 | | 4 | 6 |  | .02* |  | .48 |
| Female | 16 | | 17 | 14 |  |  |  |  |
| Race |  | |  |  |  |  |  |  |
| White | 25 | | 11 | 6 |  | .16 |  | .28 |
| Black | 1 | | 4 | 5 |  |  |  |  |
| Asian | 2 | | 0 | 1 |  |  |  |  |
| American Indian/Alaska Native | 3 | | 3 | 1 |  |  |  |  |
| Biracial/Multiracial | 3 | | 3 | 7 |  |  |  |  |
| Diagnosis |  | |  |  |  |  |  |  |
| MDD | 0 | | 15 | 13 |  | <.001*** |  | .99 |
| Anxiety Disorders (SAD, GAD) | 0 | | 16 | 11 |  | <.001*** |  | .34 |
| PTSD | 0 | | 4 | 0 |  | .01* |  | .11 |
| ADHD (self-reported) | 0 | | 3 | 5 |  | .04* |  | .43 |
|  |  | |  |  | *W* | *p* | *W* | *p* |
| Education |  | |  |  |  |  |  |  |
| 6th grade | 0 | | 0 | 1 | 355 | .97 | 203 | .86 |
| 7th grade | 4 | | 5 | 2 |  |  |  |  |
| 8th grade | 13 | | 5 | 5 |  |  |  |  |
| 9th grade | 8 | | 4 | 5 |  |  |  |  |
| 10th grade | 5 | | 2 | 5 |  |  |  |  |
| 11th grade | 3 | | 5 | 1 |  |  |  |  |
| 13th grade | 1 | | 0 | 0 |  |  |  |  |
| 15th grade | 0 | | 0 | 1 |  |  |  |  |
| Family Income |  | |  |  |  |  |  |  |
| $0–$49,999 | 1 | | 10 | 9 | 392 | .002** | 114.5 | .87 |
| $50,000–$99,999 | 11 | | 3 | 3 |  |  |  |  |
| $100,000–$149,999 | 9 | | 2 | 0 |  |  |  |  |
| $150,000–$199,999 | 5 | | 3 | 0 |  |  |  |  |
| >$200,000 | 4 | | 0 | 2 |  |  |  |  |

Abbreviations: MDD: major depression disorder; PTSD: Post-traumatic stress disorder

HC: healthy control; ELA, early-life adversity; nf: neurofeedback.

Table S2. Self-reported symptom measures for HC-NF, ELA-NF, and ELA-SHAM.

| Measures | HC-NF (n=34) | ELA-NF (n=21) | ELA-SHAM (n=20) | HC-NF vs ELA-NF | | ELA-NF vs ELA-SHAM | |
| --- | --- | --- | --- | --- | --- | --- | --- |
|  |  |  |  | *t* | *p* | *t* | *p* |
| Childhood Trauma Questionnaire (CTQ) |  |  |  |  |  |  |  |
| emotional abuse | 6.58(1.99) | 13.9(4.99) | 11.85(4.47) | -6.41 | <.001*** | 1.39 | .17 |
| physical abuse | 5.58(1.05) | 7.95(2.80) | 7.8(2.91) | -3.71 | <.001*** | 0.17 | .87 |
| sexual abuse | 5.06(.24) | 6.52(3.39) | 5.9(2.73) | -1.98 | 0.06 | 0.65 | .52 |
| emotional neglect | 6.91(2.22) | 13.1(3.81) | 11.7(4.34) | -6.75 | <.001*** | 1.09 | .28 |
| physical neglect | 5.41(0.78) | 8.24(2.32) | 7.8(3.09) | -5.39 | <.001*** | 0.51 | .61 |
| Maltreatment and Abuse Chronology of Exposure (MACE) |  |  |  |  |  |  |  |
| parental emotional neglect | 0.24(.61) | 1.48(1.21) | 1.00(1.12) | -4.38 | <.001*** | 1.31 | .20 |
| parental non-verbal emotional abuse | 0.21(.41) | 2.76(1.7) | 2.25(1.55) | -6.77 | <.001*** | 1.01 | 0.32 |
| parental physical maltreatment | 0.5(.71) | 2.39(1.47) | 1.89(1.74) | -5.50 | <.001*** | 1.16 | .26 |
| parental verbal abuse | 0.35(.81) | 2.43(1.60) | 2.05(1.54) | -5.52 | <.001*** | 0.77 | .44 |
| peer emotional abuse | 0.88(1.15) | 3.19(1.60) | 2.391.63) | -5.76 | <.001*** | 1.77 | .09 |
| peer physical bullying | 0.29(0.84) | 0.43(0.75) | 0.2(0.52) | -0.62 | .54 | 1.14 | .26 |
| physical neglect | 0.17(0.63) | 0.48(0.75) | 0.95(1.43) | -1.53 | .13 | -1.32 | .20 |
| sexual abuse | 0.00 | 0.10(0.44) | 0.05(0.22) | -1.00 | .33 | 0.42 | .68 |
| witnessing interparental violence | 0.17(0.52) | 1.71(1.79) | 1.75(1.89) | -3.83 | <.001*** | -0.06 | .95 |
| witnessing violence to siblings | 0.12(0.54) | 0.71(0.86) | 0.35(0.81) | -2.89 | <.01** | 1.41 | .17 |
| PROMIS Anxiety | 44.77 (8.35) | 58.89 (3.86) | 56.11 (6.67) | 8.54 | < .001*** | 1.63 | .11 |
| PROMIS Depression | 45.43 (8.42) | 62.05 (5.72) | 59.79 (6.45) | 8.79 | < .001*** | 1.2 | .24 |

Abbreviations: PROMIS: Patient-Reported Outcomes Measurement Information System

HC: healthy control; ELA, early-life adversity; NF : neurofeedback.

Table S3. Unadjusted means, standard deviations, effect sizes, and main analyses of task ratings and symptom measures across timepoints for healthy and ELA participants in the neurofeedback condition.

| HC-NF vs ELA-NF | Estimate | SE | *t* | *p* | Cohen’s d |  |
| --- | --- | --- | --- | --- | --- | --- |
| Follow Instructions |  |  |  |  |  |  |
| Group | 1.12 | 0.58 | 1.95 | 0.05 | -- |  |
| Run |  |  |  |  |  |  |
| NF-1 | 1.64 | 0.44 | 3.68 | <0.001*** | 0.55 |  |
| NF-2 | 1.34 | 0.44 | 3.08 | 0.002** | 0.46 |  |
| NF-3 | 1.06 | 0.45 | 2.36 | 0.02* | 0.35 |  |
| TRS | 0.68 | 0.43 | 1.58 | 0.12 | 0.24 |  |
| Group * Run |  |  |  |  |  |  |
| NF-1 | -1.06 | 0.53 | -1.99 | 0.047* | -0.30 |  |
| NF-2 | -0.87 | 0.52 | -1.67 | 0.10 | -0.25 |  |
| NF-3 | -0.71 | 0.53 | -1.34 | 0.18 | -0.20 |  |
| TRS | -0.59 | 0.51 | -1.16 | 0.25 | -0.17 |  |
| Age | 0.11 | 0.16 | 0.64 | 0.53 | 0.19 |  |
| Sex | 0.06 | 0.42 | 0.15 | 0.88 | 0.05 |  |
| Describe |  |  |  |  |  |  |
| Group | 1.20 | 0.53 | 2.28 | 0.02* | -- |  |
| Run |  |  |  |  |  |  |
| NF-1 | -0.17 | 0.44 | -0.40 | 0.69 | -0.06 |  |
| NF-2 | -0.18 | 0.43 | -0.41 | 0.69 | -0.06 |  |
| NF-3 | 0.36 | 0.44 | 0.81 | 0.42 | 0.12 |  |
| TRS | -0.20 | 0.42 | -0.47 | 0.64 | -0.07 |  |
| Group * Run |  |  |  |  |  |  |
| NF-1 | 0.33 | 0.53 | 0.62 | 0.54 | 0.09 |  |
| NF-2 | 0.63 | 0.52 | 1.21 | 0.23 | 0.18 |  |
| NF-3 | -0.25 | 0.52 | -0.48 | 0.63 | -0.07 |  |
| TRS | 0.39 | 0.51 | 0.77 | 0.44 | 0.12 |  |
| Age | 0.17 | 0.14 | 1.22 | 0.23 | 0.36 |  |
| Sex | 0.67 | 0.36 | 1.87 | 0.07 | 0.56 |  |
| Clear Mind |  |  |  |  |  |  |
| Group | 0.21 | 0.66 | 0.32 | 0.75 | -- |  |
| Run |  |  |  |  |  |  |
| NF-1 | 0.43 | 0.50 | 0.87 | 0.39 | 0.13 |  |
| NF-2 | 0.002 | 0.48 | -0.01 | 0.99 | -0.0006 |  |
| NF-3 | 0.32 | 0.50 | 0.63 | 0.53 | 0.09 |  |
| TRS | -0.40 | 0.47 | -0.83 | 0.41 | -0.12 |  |
| Group * Run |  |  |  |  |  |  |
| NF-1 | -0.01 | 0.59 | -0.03 | 0.98 | -0.003 |  |
| NF-2 | 0.71 | 0.58 | 1.22 | 0.22 | 0.18 |  |
| NF-3 | 0.38 | 0.59 | 0.65 | 0.52 | 0.10 |  |
| TRS | 0.92 | 0.57 | 1.61 | 0.11 | 0.24 |  |
| Age | 0.07 | 0.19 | 0.40 | 0.69 | 0.12 |  |
| Sex | -0.66 | 0.48 | -1.38 | 0.18 | -0.41 |  |
| Focus on Breath |  |  |  |  |  |  |
| Group | 0.20 | 0.57 | 0.35 | 0.73 | -- |  |
| Run |  |  |  |  |  |  |
| NF-1 | -0.28 | 0.60 | -0.47 | 0.64 | -0.07 |  |
| NF-2 | -0.28 | 0.59 | -0.47 | 0.64 | -0.07 |  |
| NF-3 | -0.17 | 0.61 | -0.29 | 0.78 | -0.04 |  |
| TRS | -0.49 | 0.58 | -0.84 | 0.40 | -0.12 |  |
| Group * Run |  |  |  |  |  |  |
| NF-1 | -0.30 | 0.72 | -0.41 | 0.68 | -0.06 |  |
| NF-2 | -0.40 | 0.71 | -0.57 | 0.57 | -0.08 |  |
| NF-3 | -0.21 | 0.72 | -0.29 | 0.77 | -0.04 |  |
| TRS | 0.01 | 0.70 | 0.02 | 0.99 | 0.00 |  |
| Age | -0.18 | 0.12 | -1.50 | 0.14 | -0.44 |  |
| Sex | -0.53 | 0.29 | -1.82 | 0.08 | -0.55 |  |
| Mind Wander |  |  |  |  |  |  |
| Group | -0.48 | 0.61 | -0.79 | 0.43 | -- |  |
| Run |  |  |  |  |  |  |
| NF-1 | 0.32 | 0.67 | 0.47 | 0.64 | 0.07 |  |
| NF-2 | -0.52 | 0.66 | -0.78 | 0.44 | -0.12 |  |
| NF-3 | 0.01 | 0.67 | 0.02 | 0.98 | 0.003 |  |
| TRS | 0.32 | 0.65 | 0.50 | 0.62 | 0.07 |  |
| Group * Run |  |  |  |  |  |  |
| NF-1 | -0.78 | 0.56 | -1.38 | 0.17 | -0.20 |  |
| NF-2 | 0.46 | 0.55 | 0.83 | 0.41 | 0.12 |  |
| NF-3 | 0.06 | 0.57 | 0.11 | 0.91 | 0.02 |  |
| TRS | 0.22 | 0.54 | 0.41 | 0.68 | 0.06 |  |
| Age | -0.22 | 0.15 | -1.44 | 0.16 | -0.42 |  |
| Sex | 0.42 | 0.38 | 1.12 | 0.27 | 0.33 |  |
| Blue Bar |  |  |  |  |  |  |
| Group | -0.45 | 0.54 | -0.83 | 0.41 | -- |  |
| Run |  |  |  |  |  |  |
| NF-2 | -0.10 | 0.49 | -0.20 | 0.84 | -0.04 |  |
| NF-3 | 0.32 | 0.52 | 0.61 | 0.54 | 0.13 |  |
| Group * Run |  |  |  |  |  |  |
| NF-2 | 0.38 | 0.60 | 0.64 | 0.52 | 0.14 |  |
| NF-3 | 0.02 | 0.62 | 0.03 | 0.98 | 0.01 |  |
| Age | 0.10 | 0.14 | 0.67 | 0.50 | 0.20 |  |
| Sex | -0.66 | 0.35 | -1.86 | 0.07 | -0.56 |  |
| Red Bar |  |  |  |  |  |  |
| Group | 0.36 | 0.58 | 0.62 | 0.54 | -- |  |
| Run |  |  |  |  |  |  |
| NF-2 | -0.11 | 0.49 | -0.22 | 0.82 | -0.05 |  |
| NF-3 | -0.49 | 0.52 | -0.95 | 0.35 | -0.20 |  |
| Group * Run |  |  |  |  |  |  |
| NF-2 | -0.29 | 0.59 | -0.50 | 0.62 | -0.11 |  |
| NF-3 | 0.18 | 0.62 | 0.29 | 0.77 | 0.06 |  |
| Age | 0.22 | 0.16 | 1.38 | 0.17 | 0.41 |  |
| Sex | -0.03 | 0.39 | -0.09 | 0.93 | -0.03 |  |
| Current Feeling |  |  |  |  |  |  |
| Group | -1.99 | 0.51 | -3.89 | <0.001*** | -- |  |
| Run |  |  |  |  |  |  |
| NF-1 | -0.15 | 0.44 | -0.33 | 0.74 | -0.05 |  |
| NF-2 | -0.38 | 0.43 | -0.88 | 0.38 | -0.13 |  |
| NF-3 | -0.88 | 0.44 | -2.00 | 0.047* | -0.30 |  |
| TRS | -0.93 | 0.42 | -2.23 | 0.03* | -0.33 |  |
| Group * Run |  |  |  |  |  |  |
| NF-1 | 0.60 | 0.52 | 1.15 | 0.25 | 0.17 |  |
| NF-2 | 0.53 | 0.51 | 1.03 | 0.30 | 0.15 |  |
| NF-3 | 0.84 | 0.52 | 1.61 | 0.11 | 0.24 |  |
| TRS | 0.97 | 0.50 | 1.94 | 0.05 | 0.29 |  |
| Age | -0.31 | 0.14 | -2.29 | 0.03* | -0.68 |  |
| Sex | -0.69 | 0.34 | -2.01 | 0.05 | -0.60 |  |
| Symptoms | Estimate | SE | *t* | *p* | Cohen’s d |  |
| Perceived Stress | | | | | | |
| Group | -10.94 | 1.66 | -6.57 | <0.001*** | -- |  |
| Timepoint |  |  |  |  |  |  |
| T2 | -1.43 | 0.85 | -1.68 | 0.10 | -0.33 |  |
| T3 | -3.00 | 0.85 | -3.52 | <0.001*** | -0.68 |  |
| Group * Timepoint |  |  |  |  |  |  |
| T2 | 1.90 | 1.08 | 1.75 | 0.08 | 0.34 |  |
| T3 | 3.41 | 1.08 | 3.15 | 0.002** | 0.61 |  |
| Age | -0.37 | 0.58 | -0.63 | 0.53 | -0.18 |  |
| Sex | -0.19 | 1.52 | -0.13 | 0.90 | -0.04 |  |
| Positive Affect |  |  |  |  |  |  |
| Group | 14.03 | 2.55 | 5.50 | <0.001*** | -- |  |
| Timepoint |  |  |  |  |  |  |
| T2 | 0.24 | 1.31 | 0.18 | 0.86 | 0.04 |  |
| T3 | 3.86 | 1.31 | 2.95 | 0.003** | 0.57 |  |
| Group * Timepoint |  |  |  |  |  |  |
| T2 | -1.36 | 1.67 | -0.81 | 0.42 | -0.16 |  |
| T3 | -4.71 | 1.67 | -2.81 | 0.005** | -0.55 |  |
| Age | 1.86 | 0.89 | 2.09 | 0.04* | 0.58 |  |
| Sex | -2.56 | 2.34 | -1.10 | 0.28 | -0.31 |  |
| Negative Affect |  |  |  |  |  |  |
| Group | -11.12 | 2.11 | -5.27 | <0.001*** | -- |  |
| Timepoint |  |  |  |  |  |  |
| T2 | -1.52 | 1.08 | -1.42 | 0.16 | -0.28 |  |
| T3 | -3.37 | 1.10 | -3.08 | 0.002** | -0.60 |  |
| Group * Timepoint |  |  |  |  |  |  |
| T2 | 1.08 | 1.37 | 0.79 | 0.43 | 0.15 |  |
| T3 | 3.37 | 1.38 | 2.44 | 0.02* | 0.48 |  |
| Age | -0.28 | 0.73 | -0.38 | 0.71 | -0.11 |  |
| Sex | -1.78 | 1.93 | -0.92 | 0.36 | -0.26 |  |
| State Mindfulness |  |  |  |  |  |  |
| Group | 8.74 | 3.93 | 2.22 | 0.03* | -- |  |
| Timepoint |  |  |  |  |  |  |
| T2 | 7.48 | 2.27 | 3.29 | 0.0013** | 0.64 |  |
| T3 | 4.76 | 2.27 | 2.10 | 0.04* | 0.41 |  |
| Group * Timepoint |  |  |  |  |  |  |
| T2 | -5.04 | 2.89 | -1.74 | 0.08 | -0.34 |  |
| T3 | -3.82 | 2.89 | -1.32 | 0.19 | -0.26 |  |
| Age | 1.91 | 1.34 | 1.43 | 0.16 | 0.40 |  |
| Sex | -0.44 | 3.52 | -0.13 | 0.90 | -0.04 |  |
| State Mindfulness of Body |  |  |  |  |  |  |
| Group | 3.20 | 1.26 | 2.54 | 0.01* | -- |  |
| Timepoint |  |  |  |  |  |  |
| T2 | 3.43 | 0.92 | 3.72 | < .001*** | 0.72 |  |
| T3 | 2.57 | 0.92 | 2.79 | 0.006** | 0.54 |  |
| Group * Timepoint |  |  |  |  |  |  |
| T2 | -2.02 | 1.17 | -1.72 | 0.09 | -0.33 |  |
| T3 | -1.95 | 1.17 | -1.67 | 0.10 | -0.32 |  |
| Age | 0.27 | 0.40 | 0.69 | 0.49 | 0.19 |  |
| Sex | -0.09 | 1.05 | -0.08 | 0.93 | -0.02 |  |
| State Mindfulness of Mind |  |  |  |  |  |  |
| Group | 5.53 | 2.86 | 1.93 | 0.06 | -- |  |
| Timepoint |  |  |  |  |  |  |
| T2 | 4.05 | 1.59 | 2.55 | 0.01* | 0.50 |  |
| T3 | 2.19 | 1.59 | 1.38 | 0.17 | 0.27 |  |
| Group * Timepoint |  |  |  |  |  |  |
| T2 | -3.02 | 2.02 | -1.50 | 0.14 | -0.29 |  |
| T3 | -1.87 | 2.02 | -0.93 | 0.36 | -0.18 |  |
| Age | 1.63 | 0.98 | 1.66 | 0.10 | 0.47 |  |
| Sex | -0.35 | 2.58 | -0.14 | 0.89 | -0.04 |  |

Abbreviations: HC, healthy control; ELA, early-life adversity; NF, Neurofeedback; OBS, Observe; TR, Transfer. T1 Pre-training and pre-MRI, T2 Post-training and post-MRI, T3 one week follow-up.

Note. Task ratings were answered following the completion of each run. Questions included "How well were you able to follow instructions on the screen? How well did the blue bar correspond with your experience of focusing on your breath? How well did the red bar correspond with the experience of your mind wandering elsewhere?" (1 = not at all; 10 = perfectly); "How easy did you find it to mentally decide whether or not the words described you? How easy did you find it to clear your mind while you were resting? How easy did you find it to focus on your breath?" (1 = not easy at all; 10 = very easy); "How much did your mind wander while you were asked to focus on your breath?" (1 = not at all; 10 = all of the time); "How do you feel right now?" (1 = perfectly calm; 10 = very anxious). Tasks questions about the blue and red neurofeedback bars were presented only after completion of NF-1, NF-2, and NF-3

Table S2. Unadjusted means, standard deviations, effect sizes, and main analyses of task ratings and symptom measures across timepoints for ELA participants: neurofeedback vs SHAM.

| ELA-NF vs ELA-SHAM | Estimate | SE | *t* | *p* | Cohen’s d |  |
| --- | --- | --- | --- | --- | --- | --- |
| Follow Instructions |  |  |  |  |  |  |
| Group | 1.40 | 0.69 | 2.03 | 0.045* | -- |  |
| Run |  |  |  |  |  |  |
| NF-1 | 1.62 | 0.53 | 3.09 | 0.002** | 0.63 |  |
| NF-2 | 1.33 | 0.52 | 2.59 | 0.01* | 0.53 |  |
| NF-3 | 1.05 | 0.53 | 1.98 | 0.05 | 0.41 |  |
| TRS | 0.67 | 0.51 | 1.33 | 0.19 | 0.27 |  |
| Group * Run |  |  |  |  |  |  |
| NF-1 | -0.73 | 0.80 | -0.91 | 0.36 | -0.19 |  |
| NF-2 | -1.01 | 0.77 | -1.30 | 0.20 | -0.27 |  |
| NF-3 | -1.31 | 0.78 | -1.67 | 0.10 | -0.35 |  |
| TRS | -1.34 | 0.76 | -1.76 | 0.08 | -0.36 |  |
| Age | 0.08 | 0.18 | 0.44 | 0.67 | 0.18 |  |
| Sex | -1.24 | 0.55 | -2.26 | 0.03* | -0.93 |  |
| Describe |  |  |  |  |  |  |
| Group | 1.29 | 0.79 | 1.64 | 0.11 | -- |  |
| Run |  |  |  |  |  |  |
| NF-1 | -0.17 | 0.49 | -0.34 | 0.73 | -0.07 |  |
| NF-2 | -0.16 | 0.48 | -0.34 | 0.74 | -0.07 |  |
| NF-3 | 0.36 | 0.49 | 0.74 | 0.46 | 0.15 |  |
| TRS | -0.19 | 0.47 | -0.40 | 0.69 | -0.08 |  |
| Group * Run |  |  |  |  |  |  |
| NF-1 | -0.79 | 0.75 | -1.06 | 0.29 | -0.22 |  |
| NF-2 | -0.79 | 0.72 | -1.10 | 0.27 | -0.23 |  |
| NF-3 | -1.15 | 0.73 | -1.59 | 0.12 | -0.33 |  |
| TRS | -0.93 | 0.71 | -1.31 | 0.19 | -0.27 |  |
| Age | 0.05 | 0.23 | 0.21 | 0.84 | 0.09 |  |
| Sex | 0.91 | 0.64 | 1.42 | 0.17 | 0.59 |  |
| Clear Mind |  |  |  |  |  |  |
| Group | 1.39 | 0.84 | 1.66 | 0.10 | -- |  |
| Run |  |  |  |  |  |  |
| NF-1 | 0.42 | 0.56 | 0.75 | 0.46 | 0.15 |  |
| NF-2 | -0.01 | 0.55 | -0.03 | 0.98 | -0.01 |  |
| NF-3 | 0.32 | 0.56 | 0.56 | 0.57 | 0.12 |  |
| TRS | -0.40 | 0.54 | -0.75 | 0.45 | -0.15 |  |
| Group * Run |  |  |  |  |  |  |
| NF-1 | -1.12 | 0.86 | -1.30 | 0.20 | -0.27 |  |
| NF-2 | -1.42 | 0.82 | -1.73 | 0.09 | -0.36 |  |
| NF-3 | -0.51 | 0.83 | -0.61 | 0.54 | -0.13 |  |
| TRS | 0.22 | 0.82 | 0.27 | 0.79 | 0.05 |  |
| Age | 0.24 | 0.24 | 1.01 | 0.32 | 0.41 |  |
| Sex | 1.03 | 0.66 | 1.56 | 0.13 | 0.64 |  |
| Focus on Breath |  |  |  |  |  |  |
| Group | 0.99 | 0.75 | 1.33 | 0.19 | -- |  |
| Run |  |  |  |  |  |  |
| NF-1 | -0.26 | 0.67 | -0.39 | 0.70 | -0.08 |  |
| NF-2 | -0.26 | 0.66 | -0.39 | 0.70 | -0.08 |  |
| NF-3 | -0.20 | 0.68 | -0.30 | 0.77 | -0.06 |  |
| TRS | -0.48 | 0.65 | -0.74 | 0.46 | -0.15 |  |
| Group * Run |  |  |  |  |  |  |
| NF-1 | -1.98 | 1.03 | -1.93 | 0.06 | -0.39 |  |
| NF-2 | -1.91 | 0.99 | -1.93 | 0.06 | -0.39 |  |
| NF-3 | -2.05 | 1.01 | -2.04 | 0.04* | -0.42 |  |
| TRS | -2.10 | 0.98 | -2.14 | 0.04* | -0.43 |  |
| Age | -0.28 | 0.14 | -1.94 | 0.06 | -0.78 |  |
| Sex | 0.32 | 0.39 | 0.83 | 0.42 | 0.34 |  |
| Mind Wander |  |  |  |  |  |  |
| Group | -0.55 | 0.78 | -0.71 | 0.48 | -- |  |
| Run |  |  |  |  |  |  |
| NF-1 | -0.77 | 0.54 | -1.43 | 0.16 | -0.29 |  |
| NF-2 | 0.47 | 0.53 | 0.88 | 0.38 | 0.18 |  |
| NF-3 | 0.07 | 0.55 | 0.12 | 0.90 | 0.03 |  |
| TRS | 0.23 | 0.52 | 0.44 | 0.66 | 0.09 |  |
| Group * Run |  |  |  |  |  |  |
| NF-1 | 0.91 | 0.83 | 1.10 | 0.27 | 0.23 |  |
| NF-2 | 0.27 | 0.79 | 0.34 | 0.74 | 0.07 |  |
| NF-3 | 0.58 | 0.80 | 0.73 | 0.47 | 0.15 |  |
| TRS | 1.51 | 0.79 | 1.91 | 0.06 | 0.39 |  |
| Age | -0.15 | 0.22 | -0.70 | 0.49 | -0.28 |  |
| Sex | -0.06 | 0.59 | -0.10 | 0.92 | -0.04 |  |
| Blue Bar |  |  |  |  |  |  |
| Group | -1.85 | 0.73 | -2.54 | 0.01* | -- |  |
| Run |  |  |  |  |  |  |
| NF-2 | -0.11 | 0.59 | -0.19 | 0.85 | -0.05 |  |
| NF-3 | 0.27 | 0.62 | 0.44 | 0.66 | 0.12 |  |
| Group * Run |  |  |  |  |  |  |
| NF-2 | 0.75 | 0.91 | 0.83 | 0.41 | 0.24 |  |
| NF-3 | 0.71 | 0.93 | 0.76 | 0.45 | 0.22 |  |
| Age | -0.14 | 0.17 | -0.81 | 0.43 | -0.33 |  |
| Sex | -0.67 | 0.46 | -1.46 | 0.16 | -0.63 |  |
| Red Bar |  |  |  |  |  |  |
| Group | 0.40 | 0.81 | 0.50 | 0.62 | -- |  |
| Run |  |  |  |  |  |  |
| NF-2 | -0.09 | 0.50 | -0.19 | 0.85 | -0.06 |  |
| NF-3 | -0.47 | 0.54 | -0.88 | 0.39 | -0.25 |  |
| Group * Run |  |  |  |  |  |  |
| NF-2 | -1.18 | 0.77 | -1.54 | 0.13 | -0.46 |  |
| NF-3 | -0.80 | 0.79 | -1.02 | 0.32 | -0.30 |  |
| Age | 0.31 | 0.24 | 1.30 | 0.21 | 0.52 |  |
| Sex | -0.89 | 0.65 | -1.35 | 0.19 | -0.56 |  |
| Current Feeling |  |  |  |  |  |  |
| Group | -1.37 | 0.75 | -1.81 | 0.08 | -- |  |
| Run |  |  |  |  |  |  |
| NF-1 | -0.14 | 0.49 | -0.29 | 0.78 | -0.06 |  |
| NF-2 | -0.37 | 0.48 | -0.78 | 0.44 | -0.16 |  |
| NF-3 | -0.87 | 0.49 | -1.78 | 0.08 | -0.37 |  |
| TRS | -0.94 | 0.47 | -2.00 | 0.048* | -0.41 |  |
| Group * Run |  |  |  |  |  |  |
| NF-1 | 0.20 | 0.75 | 0.27 | 0.79 | 0.06 |  |
| NF-2 | -0.13 | 0.72 | -0.18 | 0.86 | -0.04 |  |
| NF-3 | 1.03 | 0.73 | 1.43 | 0.16 | 0.30 |  |
| TRS | 0.10 | 0.71 | 0.14 | 0.89 | 0.03 |  |
| Age | -0.30 | 0.22 | -1.36 | 0.19 | -0.56 |  |
| Sex | -0.66 | 0.60 | -1.11 | 0.28 | -0.46 |  |
| Measure | Estimate | SE | *t* | *p* | Cohen’s d |  |
| Perceived Stress | | | | | | |
| Group | -4.40 | 2.15 | -2.04 | 0.045* | -- |  |
| Timepoint |  |  |  |  |  |  |
| T2 | -1.43 | 1.24 | -1.15 | 0.25 | -0.26 |  |
| T3 | -3.00 | 1.24 | -2.42 | 0.02* | -0.55 |  |
| Group * Timepoint |  |  |  |  |  |  |
| T2 | 0.98 | 1.77 | 0.55 | 0.58 | 0.13 |  |
| T3 | 2.85 | 1.77 | 1.61 | 0.11 | 0.36 |  |
| Age | -0.19 | 0.73 | -0.26 | 0.80 | -0.08 |  |
| Sex | -1.54 | 2.21 | -0.70 | 0.49 | -0.23 |  |
| Positive Affect |  |  |  |  |  |  |
| Group | 3.22 | 3.05 | 1.06 | 0.29 | -- |  |
| Timepoint | 0.24 | 1.67 | 0.14 | 0.89 | 0.03 |  |
| T2 | 3.86 | 1.67 | 2.31 | 0.02* | 0.53 |  |
| T3 |  |  |  |  |  |  |
| Group * Timepoint |  |  |  |  |  |  |
| T2 | -1.39 | 2.39 | -0.58 | 0.56 | -0.13 |  |
| T3 | -3.60 | 2.44 | -1.48 | 0.14 | -0.34 |  |
| Age | 0.28 | 1.05 | 0.27 | 0.79 | 0.09 |  |
| Sex | 2.85 | 3.19 | 0.90 | 0.38 | 0.29 |  |
| Negative Affect |  |  |  |  |  |  |
| Group | -2.29 | 2.87 | -0.80 | 0.43 | -- |  |
| Timepoint |  |  |  |  |  |  |
| T2 | -1.52 | 1.65 | -0.93 | 0.36 | -0.21 |  |
| T3 | -3.41 | 1.68 | -2.03 | 0.045* | -0.46 |  |
| Group * Timepoint |  |  |  |  |  |  |
| T2 | 1.57 | 2.36 | 0.67 | 0.51 | 0.15 |  |
| T3 | 2.07 | 2.40 | 0.86 | 0.39 | 0.20 |  |
| Age | -0.95 | 0.98 | -0.98 | 0.33 | -0.32 |  |
| Sex | -1.50 | 2.96 | -0.51 | 0.62 | -0.17 |  |
| State Mindfulness |  |  |  |  |  |  |
| Group | 2.22 | 4.72 | 0.47 | 0.64 | -- |  |
| Timepoint |  |  |  |  |  |  |
| T2 | 7.48 | 2.61 | 2.87 | 0.01* | 0.65 |  |
| T3 | 4.76 | 2.61 | 1.83 | 0.07 | 0.41 |  |
| Group * Timepoint |  |  |  |  |  |  |
| T2 | -0.48 | 3.73 | -0.13 | 0.90 | -0.03 |  |
| T3 | -0.01 | 3.73 | 0.00 | 1.00 | -0.0007 |  |
| Age | 0.33 | 1.62 | 0.20 | 0.84 | 0.07 |  |
| Sex | 2.24 | 4.90 | 0.46 | 0.65 | 0.15 |  |
| State Mindfulness of Body |  |  |  |  |  |  |
| Group | 2.53 | 1.54 | 1.64 | 0.10 | -- |  |
| Timepoint |  |  |  |  |  |  |
| T2 | 3.43 | 1.18 | 2.91 | 0.004* | 0.66 |  |
| T3 | 2.57 | 1.18 | 2.18 | 0.03* | 0.49 |  |
| Group * Timepoint |  |  |  |  |  |  |
| T2 | -1.23 | 1.69 | -0.73 | 0.47 | -0.17 |  |
| T3 | -0.62 | 1.69 | -0.37 | 0.71 | -0.08 |  |
| Age | -0.02 | 0.46 | -0.04 | 0.97 | -0.01 |  |
| Sex | -0.15 | 1.39 | -0.11 | 0.91 | -0.04 |  |
| State Mindfulness of Mind |  |  |  |  |  |  |
| Group | -0.31 | 3.47 | -0.09 | 0.93 | -- |  |
| Timepoint |  |  |  |  |  |  |
| T2 | -0.31 | 3.47 | -0.09 | 0.93 | 0.52 |  |
| T3 | 4.05 | 1.77 | 2.29 | 0.02* | 0.28 |  |
| Group * Timepoint |  |  |  |  |  |  |
| T2 | 0.75 | 2.53 | 0.30 | 0.77 | 0.07 |  |
| T3 | 0.61 | 2.53 | 0.24 | 0.81 | 0.05 |  |
| Age | 0.35 | 1.21 | 0.29 | 0.78 | 0.09 |  |
| Sex | 2.40 | 3.68 | 0.65 | 0.52 | 0.21 |  |

Abbreviations: HC, healthy control; ELA, early-life adversity; NF, Neurofeedback; OBS, Observe; TR, Transfer. T1 Pre-training and pre-MRI, T2 Post-training and post-MRI, T3 one week follow-up.

Note. Task ratings were answered following the completion of each run. Questions included "How well were you able to follow instructions on the screen? How well did the blue bar correspond with your experience of focusing on your breath? How well did the red bar correspond with the experience of your mind wandering elsewhere?" (1 = not at all; 10 = perfectly); "How easy did you find it to mentally decide whether or not the words described you? How easy did you find it to clear your mind while you were resting? How easy did you find it to focus on your breath?" (1 = not easy at all; 10 = very easy); "How much did your mind wander while you were asked to focus on your breath?" (1 = not at all; 10 = all of the time); "How do you feel right now?" (1 = perfectly calm; 10 = very anxious). Tasks questions about the blue and red neurofeedback bars were presented only after completion of NF-1, NF-2, and NF-3

| Table S3. Post hoc comparisons for task ratings and symptom measures across timepoints for healthy and ELA participants in the neurofeedback condition | | | | |
| --- | --- | --- | --- | --- |
| Task ratings | Estimate | Std. Error | z statistic | p value |
| Describe | | | | |
| Group | | | | |
| HC: ELA | 1.20 | 0.53 | 2.28 | 0.02* |
| Feeling |  |  |  |  |
| Group |  |  |  |  |
| HC: ELA | -1.99 | 0.51 | -3.89 | <0.001*** |
| Follow instruction |  |  |  |  |
| Timepoints |  |  |  |  |
| NF-1:OBS | 1.64 | 0.44 | 3.68 | 0.002** |
| NF-2:OBS | 1.34 | 0.44 | 3.08 | 0.02* |
| NF-3:OBS | 1.06 | 0.45 | 2.36 | 0.13 |
| TR:OBS | 0.67 | 0.43 | 1.58 | 0.51 |
| NF-2:NF-1 | -0.30 | 0.43 | -0.69 | 0.96 |
| NF-3:NF-1 | -0.58 | 0.46 | -1.28 | 0.70 |
| TR:NF-1 | -0.96 | 0.43 | -2.26 | 0.16 |
| NF-3:NF-2 | -0.28 | 0.45 | -0.64 | 0.97 |
| TR:NF-2 | -0.67 | 0.42 | -1.60 | 0.50 |
| TR:NF-3 | -0.38 | 0.44 | -0.87 | 0.91 |
| Symptoms | Estimate | Std. Error | z statistic | p value |
| Positive affect | | | | |
| Group |  |  |  |  |
| HC: ELA | 14.03 | 2.55 | 5.50 | <0.001*** |
| Timepoints |  |  |  |  |
| Post-training: baseline | 0.24 | 1.31 | 0.18 | 0.98 |
| One-week follow up:baseline | 3.86 | 1.31 | 2.95 | 0.01* |
| One-week follow up: post-training | 3.62 | 1.31 | 2.76 | 0.02* |
| Group_pairwise:timepoint_pairwise | |  |  |  |
| ELA-HC:baseline-post-training | -1.36 | 1.67 | -0.81 | 0.42 |
| ELA-HC:baseline-one-week follow up | -4.71 | 1.67 | -2.81 | 0.01* |
| ELA-HC:post-training-one-week follow up | -3.35 | 1.67 | -2.00 | 0.05 |
| Negative affect | | | | |
| Group |  |  |  |  |
| HC: ELA | -11.12 | 2.11 | -5.27 | <0.001*** |
| Timepoints |  |  |  |  |
| Post-training: baseline | -1.52 | 1.08 | -1.42 | 0.33 |
| One-week follow up:baseline | -3.37 | 1.10 | -3.08 | 0.01* |
| One-week follow up: post-training | -1.85 | 1.10 | -1.69 | 0.21 |
| Group_pairwise:timepoint_pairwise | | |  |  |
| ELA-HC:baseline-post-training | 1.08 | 1.37 | 0.79 | 0.43 |
| ELA-HC:baseline-one-week follow up | 3.37 | 1.38 | 2.44 | 0.02* |
| ELA-HC:post-training-one-week follow up | 2.29 | 1.38 | 1.66 | 0.10 |
| Perceived stress |  |  |  |  |
| Group |  |  |  |  |
| HC: ELA | -10.94 | 1.66 | -6.57 | <0.001*** |
| Timepoints |  |  |  |  |
| Post-training: baseline | -1.43 | 0.85 | -1.68 | 0.21 |
| One-week follow up:baseline | -3.00 | 0.85 | -3.52 | 0.001** |
| One-week follow up: post-training | -1.57 | 0.85 | -1.85 | 0.15 |
| Group_pairwise:timepoint_pairwise | | |  |  |
| ELA-HC:baseline-post-training | 1.90 | 1.08 | 1.75 | 0.08 |
| ELA-HC:baseline-one-week follow up | 3.41 | 1.08 | 3.15 | 0.002** |
| ELA-HC:post-training-one-week follow up | 1.51 | 1.08 | 1.40 | 0.17 |
| State mindfulness of body | |  |  |  |
| Group |  |  |  |  |
| HC:ELA | 3.20 | 1.26 | 2.54 | 0.01* |
| Timepoint |  |  |  |  |
| Post-training: baseline | 3.43 | 0.92 | 3.72 | <0.001*** |
| One-week follow up:baseline | 2.57 | 0.92 | 2.79 | 0.01* |
| One-week follow up: post-training | -0.86 | 0.92 | -0.93 | 0.62 |
| State mindfulness of mind | |  |  |  |
| Timepoint |  |  |  |  |
| Post-training: baseline | 4.05 | 1.59 | 2.55 | 0.03 |
| One-week follow up:baseline | 2.19 | 1.59 | 1.38 | 0.35 |
| One-week follow up: post-training | -1.86 | 1.59 | -1.17 | 0.47 |

Abbreviations: HC, healthy control; ELA, early-life adversity; NF, Neurofeedback; OBS, Observe; TR, Transfer. T1 Pre-training and pre-MRI, T2 Post-training and post-MRI, T3 one week follow-up.

Note. Task ratings were answered following the completion of each run. Questions included "How well were you able to follow instructions on the screen? How well did the blue bar correspond with your experience of focusing on your breath? How well did the red bar correspond with the experience of your mind wandering elsewhere?" (1 = not at all; 10 = perfectly); "How easy did you find it to mentally decide whether or not the words described you? How easy did you find it to clear your mind while you were resting? How easy did you find it to focus on your breath?" (1 = not easy at all; 10 = very easy); "How much did your mind wander while you were asked to focus on your breath?" (1 = not at all; 10 = all of the time); "How do you feel right now?" (1 = perfectly calm; 10 = very anxious). Tasks questions about the blue and red neurofeedback bars were presented only after completion of NF-1, NF-2, and NF-3

| Table S4. Post hoc comparisons for task ratings and symptom measures across timepoints for ELA participants: neurofeedback vs SHAM. | | | | |
| --- | --- | --- | --- | --- |
| Task ratings | Estimate | Std. Error | z statistic | p value |
| Follow instruction |  |  |  |  |
| Group |  |  |  |  |
| SHAM: NF | 1.40 | 0.69 | 2.03 | 0.04* |
| Timepoints |  |  |  |  |
| NF-1:OBS | 1.62 | 0.53 | 3.09 | 0.02* |
| NF-2:OBS | 1.33 | 0.52 | 2.59 | 0.07 |
| NF-3:OBS | 1.05 | 0.53 | 1.98 | 0.27 |
| TR:OBS | 0.67 | 0.51 | 1.33 | 0.67 |
| NF-2:NF-1 | -0.29 | 0.51 | -0.57 | 0.98 |
| NF-3:NF-1 | -0.57 | 0.54 | -1.06 | 0.83 |
| TR:NF-1 | -0.95 | 0.50 | -1.89 | 0.32 |
| NF-3:NF-2 | -0.28 | 0.53 | -0.54 | 0.98 |
| TR:NF-2 | -0.66 | 0.49 | -1.34 | 0.67 |
| TR:NF-3 | -0.38 | 0.52 | -0.74 | 0.95 |
| Blue bar | | | | |
| Group | | | | |
| SHAM: NF | -1.85 | 0.73 | -2.54 | 0.01* |
| Symptoms | Estimate | Std. Error | z statistic | p value |
| Perceived stress | | | | |
| Group |  |  |  |  |
| SHAM: NF | -4.40 | 2.15 | -2.04 | 0.04* |
| Positive affect | | | | |
| Timepoints |  |  |  |  |
| Post-training: baseline | 0.24 | 1.67 | 0.14 | 0.99 |
| One-week follow up:baseline | 3.86 | 1.67 | 2.31 | 0.05 |
| One-week follow up: post-training | 3.62 | 1.67 | 2.17 | 0.08 |
| State mindfulness of body | |  |  |  |
| Timepoint |  |  |  |  |
| Post-training: baseline | 3.43 | 1.18 | 2.91 | 0.01* |
| One-week follow up:baseline | 2.57 | 1.18 | 2.18 | 0.07 |
| One-week follow up: post-training | -0.86 | 1.18 | -0.73 | 0.75 |

Abbreviations: HC, healthy control; ELA, early-life adversity; NF, Neurofeedback; OBS, Observe; TR, Transfer. T1 Pre-training and pre-MRI, T2 Post-training and post-MRI, T3 one week follow-up.

Note. Task ratings were answered following the completion of each run. Questions included "How well were you able to follow instructions on the screen? How well did the blue bar correspond with your experience of focusing on your breath? How well did the red bar correspond with the experience of your mind wandering elsewhere?" (1 = not at all; 10 = perfectly); "How easy did you find it to mentally decide whether or not the words described you? How easy did you find it to clear your mind while you were resting? How easy did you find it to focus on your breath?" (1 = not easy at all; 10 = very easy); "How much did your mind wander while you were asked to focus on your breath?" (1 = not at all; 10 = all of the time); "How do you feel right now?" (1 = perfectly calm; 10 = very anxious). Tasks questions about the blue and red neurofeedback bars were presented only after completion of NF-1, NF-2, and NF-3

Table S5. Consensus on the Reporting and Experimental Design of clinical and cognitive-behavioural Neurofeedback studies (CRED-nf) best practices checklist 2020.

| **Domain** | **Item #** | **Checklist item** | **Reported on page #** |
| --- | --- | --- | --- |
| **Pre-experiment** | | | |
|  | 1a | Pre-register experimental protocol and planned analyses | 11 |
|  | 1b | Justify sample size | n/a |
| **Control groups** | | | |
|  | 2a | Employ control group(s) or control condition(s) | 11 |
|  | 2b | When leveraging experimental designs where a double-blind is possible, use a double-blind | n/a |
|  | 2c | Blind those who rate the outcomes, and when possible, the statisticians involved | n/a |
|  | 2d | Examine to what extent participants and experimenters remain blinded | n/a |
|  | 2e | In clinical efficacy studies, employ a standard-of-care intervention group as a benchmark for improvement | n/a |
| **Control measures** | | | |
|  | 3a | Collect data on psychosocial factors | 13 |
|  | 3b | Report whether participants were provided with a strategy | 11 |
|  | 3c | Report the strategies participants used | 11 |
|  | 3d | Report methods used for online-data processing and artifact correction | 13-14 |
|  | 3e | Report condition and group effects for artifacts | n/a |
| **Feedback specifications** | | | |
|  | 4a | Report how the online-feature extraction was defined | 14 |
|  | 4b | Report and justify the reinforcement schedule | n/a |
|  | 4c | Report the feedback modality and content | 12 |
|  | 4d | Collect and report all brain activity variable(s) and/or contrasts used for feedback, as displayed to experimental participants | 11-12 |
|  | 4e | Report the hardware and software used | 14-15 |
| **Outcome measures** | | | |
| Brain | 5a | Report neurofeedback regulation success based on the feedback signal | 12-13 |
|  | 5b | Plot within-session and between-session regulation blocks of feedback variable(s), as well as pre-to-post resting baselines or contrasts | n/a |
|  | 5c | Statistically compare the experimental condition/group to the control condition(s)/group(s) (not only each group to baseline measures) | 15-20 |
| Behaviour | 6a | Include measures of clinical or behavioural significance, defined a priori, and describe whether they were reached | 9-10 |
|  | 6b | Run correlational analyses between regulation success and behavioural outcomes | n/a |
| **Data storage** | | |  |
|  | 7a | Upload all materials, analysis scripts, code, and raw data used for analyses, as well as final values, to an open access data repository, when feasible | n/a |

Note: Darker shaded boxes represent *Essential* checklist items; lightly shaded boxes represent *Encouraged* checklist items. We recommend using this checklist in conjunction with the standardized CRED-nf online tool ([rtfin.org/CREDnf](http://www.rtfin.org/CREDnf)) and the CRED-nf article, which explains the motivation behind this checklist and provides details regarding many of the checklist items.

# Supplementary Figures

Figure S1. CONSORT Diagram. Flow diagram graphically describes the design of the study: enrolment, intervention, and data analysis. Abbreviations: ELA, early-life adversity; NAMT: neurofeedback augmented mindfulness training

Figure S2

**Figure S2.** Barplot of the signal variance ratio (R^2 value) explained by the physiological noise regressors (RETROICOR) for the real-time processed and offline processed signals in the PCC region.

Figure S3. Abbreviations: HC, healthy control; ELA, early-life adversity; NF, Neurofeedback; OBS, Observe; TR, Transfer. **p* <.05, ***p*<.01, *** *p*<.001

Top Panel: participant reported task ratings for each experimental run. Task ratings were answered following the completion of each run. Questions included "How well were you able to follow instructions on the screen? How well did the blue bar correspond with your experience of focusing on your breath? How well did the red bar correspond with the experience of your mind wandering elsewhere?" (1 = not at all; 10 = perfectly); "How easy did you find it to mentally decide whether or not the words described you? How easy did you find it to clear your mind while you were resting? How easy did you find it to focus on your breath?" (1 = not easy at all; 10 = very easy); "How much did your mind wander while you were asked to focus on your breath?" (1 = not at all; 10 = all of the time); "How do you feel right now?" (1 = perfectly calm; 10 = very anxious). Tasks questions about the blue and red neurofeedback bars were presented only after completion of NF-1, NF-2, and NF-3.

Figure S4. Abbreviations: HC, healthy control; ELA, early-life adversity; NF, Neurofeedback; OBS, Observe; TR, Transfer. **p* <.05, ***p*<.01, *** *p*<.001

Top Panel: participant reported task ratings for each experimental run. Task ratings were answered following the completion of each run. Questions included "How well were you able to follow instructions on the screen? How well did the blue bar correspond with your experience of focusing on your breath? How well did the red bar correspond with the experience of your mind wandering elsewhere?" (1 = not at all; 10 = perfectly); "How easy did you find it to mentally decide whether or not the words described you? How easy did you find it to clear your mind while you were resting? How easy did you find it to focus on your breath?" (1 = not easy at all; 10 = very easy); "How much did your mind wander while you were asked to focus on your breath?" (1 = not at all; 10 = all of the time); "How do you feel right now?" (1 = perfectly calm; 10 = very anxious). Tasks questions about the blue and red neurofeedback bars were presented only after completion of NF-1, NF-2, and NF-3.


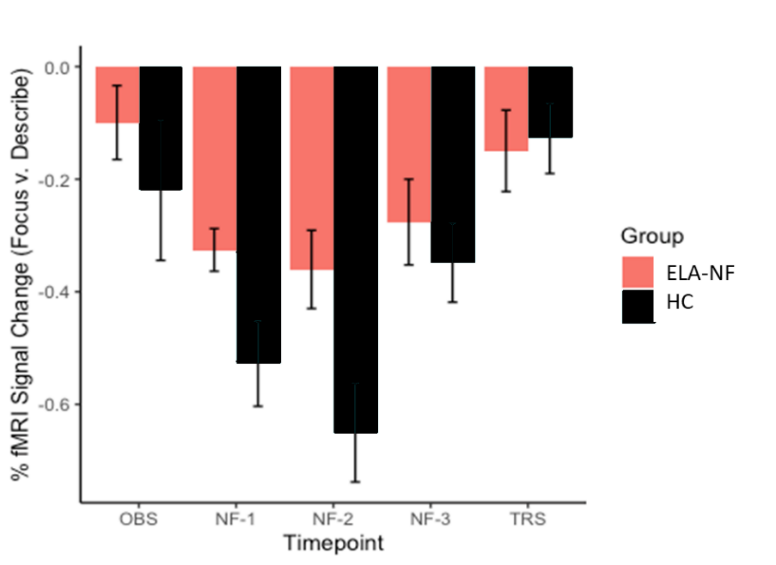


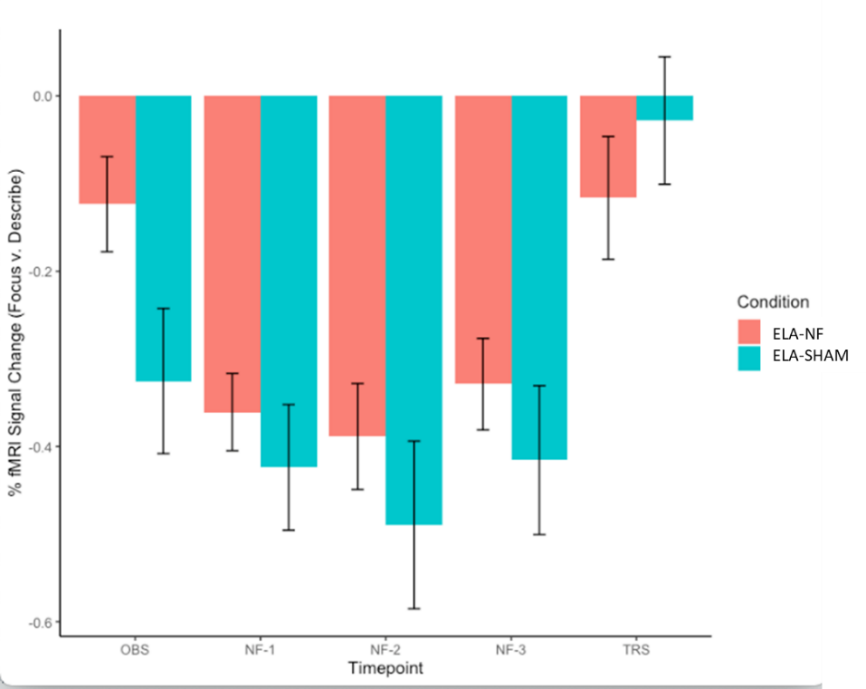


Figure S5. fMRI percent signal change for the Focus-on-Breath vs. Describe contrast across runs for HC and ELA (top), and ELA-NF and ELA-SHAM (bottom). Abbreviations: HC, healthy control; ELA, early-life adversity; NF, Neurofeedback; OBS, Observe; TR, Transfer.


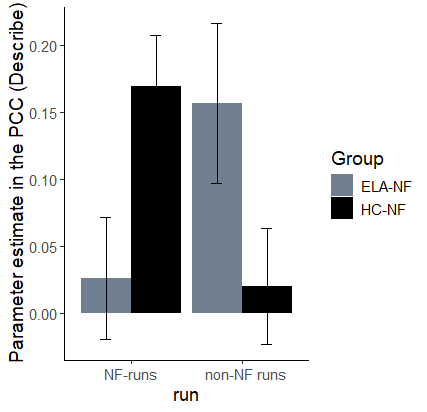

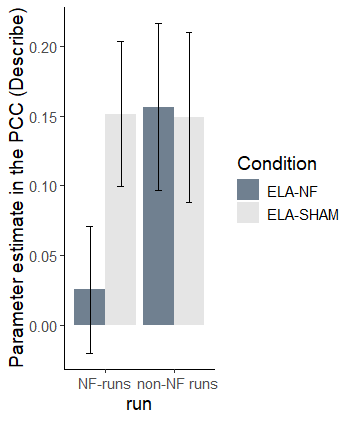


Figure S6. fMRI percent signal change for the Describe across runs for HC and ELA (left), and ELA-NF and ELA-SHAM (right). Abbreviations: HC, healthy control; ELA, early-life adversity; NF, Neurofeedback; OBS, Observe; TR, Transfer.

Figure S7. Whole-brain, voxel-wise activation map for Focus-on-Breath vs. Describe condition during neurofeedback runs for ELA-NF vs HC-NF (top) and ELA-NF vs ELA-SHAM (Bottom).

Figure S8. Real-time neurofeedback functional connectivity correlation map during neurofeedback runs for HC-NF vs ELA-NF (top) and ELA-NF vs ELA-SHAM (bottom).

References

1. Kuplicki R, Touthang J, Al Zoubi O, et al. Common data elements, scalable data management infrastructure, and analytics workflows for large-scale neuroimaging studies. *Frontiers in psychiatry*. 2021;12:682495.

2. Sheehan DV, Sheehan KH, Shytle RD, et al. Reliability and validity of the mini international neuropsychiatric interview for children and adolescents (MINI-KID). *The Journal of clinical psychiatry*. 2010;71(3):17393.

3. Brewer JA, Worhunsky PD, Gray JR, Tang Y-Y, Weber J, Kober H. Meditation experience is associated with differences in default mode network activity and connectivity. *Proceedings of the National Academy of Sciences*. 2011;108(50):20254-20259.

4. Garrison KA, Scheinost D, Worhunsky PD, et al. Real-time fMRI links subjective experience with brain activity during focused attention. *Neuroimage*. 2013;81:110-118.

5. Misaki M, Barzigar N, Zotev V, Phillips R, Cheng S, Bodurka J. Real-time fMRI processing with physiological noise correction–Comparison with off-line analysis. *Journal of neuroscience methods*. 2015;256:117-121.

6. Kelley WM, Macrae CN, Wyland CL, Caglar S, Inati S, Heatherton TF. Finding the self? An event-related fMRI study. *Journal of cognitive neuroscience*. 2002;14(5):785-794.

7. Pruessmann KP, Weiger M, Scheidegger MB, Boesiger P. SENSE: sensitivity encoding for fast MRI. *Magnetic Resonance in Medicine: An Official Journal of the International Society for Magnetic Resonance in Medicine*. 1999;42(5):952-962.

8. Kirlic N, Cohen Z, Tsuchiyagaito A, et al. Self-Regulation of the Posterior Cingulate Cortex with Real-Time fMRI Neurofeedback in Healthy Adolescents 2021;

9. Wang S, Taren AA, Smith DV. Functional Parcellation of the Default Mode Network: A Large-Scale Meta-Analysis. *bioRxiv*. 2018:225375.

10. Misaki M, Bodurka JA. The impact of real-time fMRI denoising on online evaluation of brain activity and functional connectivity. *Journal of Neural Engineering*. 2021;

11. Glover GH, Li TQ, Ress D. Image‐based method for retrospective correction of physiological motion effects in fMRI: RETROICOR. *Magnetic Resonance in Medicine: An Official Journal of the International Society for Magnetic Resonance in Medicine*. 2000;44(1):162-167.

12. Zotev V, Phillips R, Yuan H, Misaki M, Bodurka J. Self-regulation of human brain activity using simultaneous real-time fMRI and EEG neurofeedback. *NeuroImage*. 2014;85:985-995.

13. Wong C-K, Zotev V, Misaki M, Phillips R, Luo Q, Bodurka J. Automatic EEG-assisted retrospective motion correction for fMRI (aE-REMCOR). *Neuroimage*. 2016;129:133.

14. Birn RM, Diamond JB, Smith MA, Bandettini PA. Separating respiratory-variation-related fluctuations from neuronal-activity-related fluctuations in fMRI. *Neuroimage*. 2006;31(4):1536-1548.

15. Chang C, Cunningham JP, Glover GH. Influence of heart rate on the BOLD signal: the cardiac response function. *Neuroimage*. 2009;44(3):857-869.

16. Avants BB, Epstein CL, Grossman M, Gee JC. Symmetric diffeomorphic image registration with cross-correlation: evaluating automated labeling of elderly and neurodegenerative brain. *Medical Image Analysis*. 2008;12(1):26-41.

17. Peirce J, Gray JR, Simpson S, et al. PsychoPy2: Experiments in behavior made easy. *Behavior research methods*. 2019;51(1):195-203.

18. Zotev V, Krueger F, Phillips R, et al. Self-regulation of amygdala activation using real-time fMRI neurofeedback. *PloS one*. 2011;6(9):e24522.

19. Birn RM, Smith MA, Jones TB, Bandettini PA. The respiration response function: the temporal dynamics of fMRI signal fluctuations related to changes in respiration. *Neuroimage*. 2008;40(2):644-654.

20. Jo HJ, Saad ZS, Simmons WK, Milbury LA, Cox RW. Mapping sources of correlation in resting state FMRI, with artifact detection and removal. *Neuroimage*. 2010;52(2):571-582.

21. Misaki M, Bodurka J. The impact of real-time fMRI denoising on online evaluation of brain activity and functional connectivity. *Journal of Neural Engineering*. 2021;18(4):046092.
